# CO_2_ flux emissions from the Earth’s most actively degassing volcanoes, 2005–2015

**DOI:** 10.1038/s41598-019-41901-y

**Published:** 2019-04-01

**Authors:** Alessandro Aiuppa, Tobias P. Fischer, Terry Plank, Philipson Bani

**Affiliations:** 1 Dipartimento DiSTeM, Università di Palermo, Palermo, Italy; 2 Department of Earth and Planetary Sciences, New Mexico University, Albuquerque, USA; 3 Lamont-Doherty Earth Observatory, Columbia University, New York, USA; 4 Laboratoire Magmas et Volcans, Université Blaise Pascal - CNRS -IRD, OPGC, Aubière, France

## Abstract

The global carbon dioxide (CO_2_) flux from subaerial volcanoes remains poorly quantified, limiting our understanding of the deep carbon cycle during geologic time and in modern Earth. Past attempts to extrapolate the global volcanic CO_2_ flux have been biased by observations being available for a relatively small number of accessible volcanoes. Here, we propose that the strong, but yet unmeasured, CO_2_ emissions from several remote degassing volcanoes worldwide can be predicted using regional/global relationships between the CO_2_/S_T_ ratio of volcanic gases and whole-rock trace element compositions (e.g., Ba/La). From these globally linked gas/rock compositions, we predict the CO_2_/S_T_ gas ratio of 34 top-degassing remote volcanoes with no available gas measurements. By scaling to volcanic SO_2_ fluxes from a global catalogue, we estimate a cumulative “unmeasured” CO_2_ output of 11.4 ± 1.1 Mt/yr (or 0.26 ± 0.02·10^12^ mol/yr). In combination with the measured CO_2_ output of 27.4 ± 3.6 Mt/yr (or 0.62 ± 0.08·10^12^ mol/yr), our results constrain the time-averaged (2005–2015) cumulative CO_2_ flux from the Earth’s 91 most actively degassing subaerial volcanoes at 38.7 ± 2.9 Mt/yr (or 0.88 ± 0.06·10^12^ mol/yr).

## Introduction

Volcanism is the primary mechanism through which carbon (C) stored in the deep Earth^1,2^ is transferred to surface environments to feed C exchanges in the atmosphere-ocean-biosphere system^3^. Over geological time, volcanic CO_2_ emissions have been a key control on atmospheric-oceanic CO_2_ levels^4–8^, ultimately regulating evolution of climate and life on our planet^9,10^.

The global volcanic CO_2_ flux in modern Earth remains inadequately known^11,12^ and, ironically, is less constrained for subaerial volcanoes than for the less-accessible mid-ocean ridges, for which the ^3^He flux^13^ or the CO_2_/Ba ratio^14^ proxies have successfully been applied. Direct volcanic CO_2_ observations at subaerial volcanoes are technically challenging from both ground^11,15^ and space^16^ due to the large atmospheric CO_2_ burden, and thus remain limited in number^17,18^_._ The volcanic CO_2_ flux can be quantified indirectly by combining simultaneous acquisitions of UV-sensed sulphur dioxide (SO_2_) fluxes^11,15,19,20^ and gas compositions (CO_2_/SO_2_ ratios), but gas observational networks are still in a developing stage^21,22^, resulting in sparse and incomplete gas catalogues^23,24^. CO_2_ flux data have so far been obtained^11,15^ for only <60 of the several hundred currently degassing Holocene volcanoes^25^. CO_2_ flux records are continuous enough only for a few (<10) volcanoes where permanent instrumentation is operating^26–29^, while sparse results (one or a few campaign-style measurements at most) are available for the remaining ~50. In addition, scarce or even no information exists for several top-ranking degassing volcanoes^30^ in remote regions of the world (e.g., Vanuatu^31^, Papua New Guinea, the Solomon arc, and the Sunda-Banda arc in Indonesia^32,33^). Attempts to extrapolate available measurements to all the subaerial degassing volcanoes have been problematic^11,23^ and require use of questionable statistical approaches^34,35^. Estimates of the global volcanic CO_2_ flux thus vary widely, from 66 to 540 Mt/yr^11,23^.

Ideally, refining the volcanic CO_2_ inventory would require a comprehensive record comprising simultaneous composition/emission measurements for all the currently active strong volcanic gas emitters globally. The top-degassing volcanic targets during 2005–2015 (Table 1) have recently been identified^30^ from satellite-based observations of the SO_2_ flux using the Ozone Mapping Instrument (OMI). Carn *et al*. (ref.^30^) identified 91 volcanoes, listed in Table 1, releasing SO_2_ at rates above the OMI detection limit of 16 tons/day. Gas CO_2_/S_T_ ratios (where S_T_ is Total Sulfur, corresponding to SO_2_ in these strongly degassing magmatic-volatile emitting volcanoes) are available for 57 out of these 91 volcanic sources^36^, from which SO_2_ fluxes can straightforwardly be converted into CO_2_ fluxes (Table 1). The remaining 34 volcanoes, however, have so far been impossible to characterise for gas composition, owing to their remoteness and/or poor accessibility, leaving their CO_2_ fluxes unconstrained.

**Table 1:** Gas composition and fluxes for the 91 strongest SO_2_ volcanic gas sources in 2005–2015 worldwide (from Carn *et al*., 2017).

	Measured volcanoes												
A	B	B	D	E	F	G	H	K	I	L = F × H	M = F × K	N	O
Group	Volcano	Country	Lat	Long	Measured SO_2_ flux (tons/day)	SD	Measured CO_2_/SO_2_ (molar)	Predicted CO_2_/SO_2_ (molar)	SD	Measured CO_2_ flux (tons/day)	Predicted CO_2_ flux	SD	Notes/Data Sources
1	Ambrym	Vanuatu	−16.25	168.12	7356	3168	1.5	—	*0*.*4*	7586	—	3843	
1	Asama	Japan	36.40	138.53	449	430	0.8	—	*0*.*2*	247	—	247	
1	Aso	Japan	32.88	131.11	628	492	1.8	—	*0*.*5*	777	—	650	
1	Augustine	USA	59.35	−153.45	73	140	1.5	—	0.7	75	—	148	
1	Avachinsky	Russia	53.25	158.83	707	619	1.2*	—	*0*.*3*	584	—	531	*Ref.^110^
1	Chikurachki + Ebeko^$^	Russia	50.33	155.46	496	469	0.9*	—	*0*.*3*	320	—	317	*Ref.^111^
1	Cleveland	USA- AK	52.83	−169.77	152	142	1.0*	—	*0*.*3*	105	—	102	*Upper limit from ref.^111^
1	Copahue	Argentina	−37.86	−71.16	341	425	0.9	—	0.3	211	—	272	
1	Dukono	Indonesia	1.68	127.88	1726	611	0.4	—	0.1	475	—	206	
1	Gareloi	USA- AK	51.79	−178.79	52	47	0.5	—	*0*.*1*	18	—	17	
1	Isluga	Chile	−19.15	−68.83	78	107	1.0	—	0.0	51	—	70	
1	Kliuchevskoi + Bezymianny^$^	Russia	56.06	160.64	580	461	1.3*	—	*0*.*4*	519	—	442	*Assumes equal flux for the 2 volcanoes
1	Krakatau	Indonesia	−6.11	105.42	303	252	0.4	—	*0*.*1*	83	—	73	
1	Kudriavy	Russia	45.39	148.84	187	103	0.9	—	*0*.*3*	116	—	72	
1	Lastarria	Argentina	−25.17	−68.50	248	62	1.6	—	0.4	273	—	96	
1	Miyake-jima	Japan	34.08	139.53	1018	934	0.7	—	*0*.*2*	490	—	471	
1	Mutnovsky + Gorely^$^	Russia	52.45	158.20	753	690	1.7*	—	*0*.*5*	880	—	847	*Assumes equal flux for the 2 volcanoes
1	Pagan	Marianas	18.14	145.79	583	547	0.8	—	*0*.*2*	321	—	311	
1	Redoubt	USA	60.49	−152.75	368	1051	1.0	—	0.2	253	—	724	
1	Sabancaya	Peru	−15.80	−71.86	87	158	1.5	—	0.1	90	—	163	
1	Sakura-jima	Japan	31.59	130.66	1056	757	0.9	—	*0*.*3*	653	—	516	
1	San Miguel	El Salvador	13.42	−88.47	88	134	1.5	—	0.2	91	—	139	
1	Santa Ana	El Salvador	13.85	−89.63	97	180	1.0	—	*0*.*3*	66	—	125	
1	Satsuma-Iojima	Japan	30.79	130.31	585	190	0.4	—	*0*.*1*	161	—	70	
1	Shishaldin	USA- AK	54.76	−163.97	347	278	1.4*	—	*0*.*4*	334	—	284	*ref.^112^
1	Shiveluch	Russia	56.64	161.34	530	284	1.3*	—	*0*.*4*	473	—	289	*gas data for Klyucheskovoy are used
1	Spurr	USA	61.30	−152.25	106	106	1.1	—	*0*.*3*	80	—	83	
1	Suwanose-jima	Japan	29.64	129.72	863	314	1.0	—	*0*.*3*	593	—	280	
1	Tokachi	Japan	43.42	142.69	135	98	0.4	—	*0*.*1*	37	—	29	
1	Turrialba + Poas	Costa Rica	10.03	−83.77	751	681	1.0 (3.4*)	—	0.8	1756	—	1644	*Mean (2002–2017) Turrialba composition from de Moor *et al*., Pers. Comm..
1	Villarrica	Chile	−39.42	−71.93	281	160	1.0	—	0.3	193	—	124	
1	Yasur	Vanuatu	−19.53	169.44	1408	563	1.6	—	0.4	1549	—	730	
2	Galeras	Colombia	1.20	−77.39	218	317	3.3	—	0.5	495	—	723	
2	Lokon-Empung	Sulawesi	1.36	124.79	204	154	3.2*	—	1.0	449	—	366	*This study
2	Masaya	Nicaragua	11.98	−86.16	867	364	2.7	—	0.7	1610	—	794	
2	Mayon	Philippines	13.26	123.69	453	274	2.4	—	*0*.*7*	747	—	501	*ref.^113^
2	Nevado del Huila	Colombia	2.93	−76.03	627	665	2.0	—	*0*.*6*	862	—	947	
2	Nevado del Ruiz	Colombia	4.90	−75.32	1074	1376	3.0	—	0.5	2215	—	2862	
2	Raung + Ijen	East Java	−8.06	114.24	631	238	2.6*	—	0.5	1111	—	472	*Uses composition of Ijen only (ref.^114^)
2	San Cristobal + Telica	Nicaragua	12.70	−87.00	621	283	3.5*	—	2.0	1494	—	1092	*Assumes equal flux for the 2 volcanoes
2	Sirung	Pantar	−8.51	124.13	373	162	3.2*	—	2.0	820	—	624	*ref.^115^
2	Soufriere Hills	Montserrat	16.72	−62.18	1296	761	3.0	—	1.1	2672	—	1851	
2	Ubinas	Peru	−16.34	−70.90	222	252	2.4	—	0.5	367	—	423	
2	White Island	New Zealand	−37.52	177.18	254	107	4.0	—	1.2	699	—	362	
3	Bromo + Semeru	Java	−7.94	112.95	775	298	4.1*	—	0.7	2184	—	920	*Uses Bromo gas composition
3	Etna	Italy	37.73	15.00	2032	517	6.5	—	2.2	9083	—	3844	
3	Merapi	Java	−7.56	110.44	32	51	4.7	—	0.5	104	—	165	
3	Popocatepétl	Mexico	19.02	−98.62	1658	893	8.2	—	7.0	9345	—	9434	
3	Stromboli	Italy	38.79	15.21	181	82	7.2	—	2.8	894	—	535	
4	Alu-Dalafilla + Erta Ale	Ethiopia	13.60	40.67	64	24	2.3*	—	0.9	99	—	56	*Uses Erta Ale comp.; ref.^116^
4	Erebus	Antarctica	−77.53	167.17	52	31	27.6*	—	*4*.*9*	983	—	612	*Ref.^117^
4	Kilauea	USA	19.42	−155.29	5019	2275	0.9*	—	*0*.*2*	2933	—	1578	*Refs^28,118^
4	Nyiragongo + Nyamuragira^&^	DR Congo	−1.41	29.20	3533	2408	6.5*	—	1.2	15790	—	11149	*Refs^119–121^
4	Piton de la Fournaise	Reunion, France	−21.23	55.71	134	162	0.3*	—	*0*.*1*	28	—	34	*Refs^122^; Di Muro, pers. comm.
N.D.	Marapi	Sumatra	−0.39	100.46	34	34	20.5*	—	1.1	480	—	485	*This study
	**“Unmeasured” volcanoes: those for which no CO**_**2**_ **gas data exist**.												
A	B	B	D	E	F	G	H	K	I	L = F × H	M × F*K	N	O
Group	Volcano	Country	Lat	Long	Measured SO_2_ flux (tons/day)	SD	Measured CO_2_/SO_2_ (molar)	Predicted CO_2_/SO_2_ (molar)	SD	Measured CO_2_ flux	Predicted CO_2_ flux (tons/day)	SD	Notes/Data Sources
1	Anatahan	Northern Mariana Islands	16.35	145.67	1335	1867	—	1.2	0.5	—	1102	1607	
2	Aoba	Vanuatu	−15.40	167.83	2870	1229	—	2.5	0.7	—	4933	2524	
2	Bagana	Papua New Guinea	−6.09	155.23	3779	886	—	2.4	0.7	—	6245	2335	
2	Barren Island	India	12.28	93.86	243	341	—	2.2*	1.3	—	372	566	*From the Sunda-Banda gas-rock association; Table S1c; Fig. 2g
2	Batu Tara + Lewotolo	Indonesia	−8.27	123.51	632	177	—	2.4*	*0*.*7*	—	1043	420	*From the Sunda-Banda gas-rock association; Table S1c; Fig. 2g
1	Bulusan	Philippines	12.77	124.05	206	199	—	1.2	0.5	—	170	179	
1	Chiginagak	USA- AK	57.14	−156.99	138	127	—	1.2	0.5	—	114	115	
2	Ebulobo	Indonesia/Nusa	−8.82	121.18	86	63	—	2.6*	1.3	—	153	137	*From the Sunda-Banda gas-rock association; Table S1c; Fig. 2g
1	Fuego + Pacaya^$^	Guatemala	14.47	−90.88	252	46	—	1.6*	0.8	—	269	139	*From the CAVA gas-rock association; Table S1a; Fig. 2a
2	Gaua	Vanuatu	−14.27	167.50	434	382	—	2.5*	0.7	—	745	688	*From the Group 2 global gas-rock association; Table S1d; Fig. 3a
4	Jebel-at-Tair	Yemen	15.55	41.83	103	295	—	6.2*	*1*.*8*	—	445	1527	*Average on non-arc volcanoes
1	Kanlaon	Philippines	10.41	123.13	70	182	—	1.2	0.5	—	57	152	
3	Karangetang	Indonesia/Sulawesi	2.78	125.40	313	85	—	5.0*	1.3	—	1069	403	*From the Sunda-Banda gas-rock association; Table S1c; Fig. 2g
1	Karymsky	Russia	54.05	159.45	912	250	—	1.2	0.5	—	752	375	
2	Kerinci	Indonesia/Sumatra	−1.70	101.26	294	99	—	2.6*	*0*.*8*	—	525	233	*From the Sunda-Banda gas-rock association; Table S1c; Fig. 2g
1	Ketoi	Russia	47.34	152.48	139	151	—	1.2	0.5	—	114	133	
1	Kizimen	Russia	55.12	160.36	711	1544	—	1.2	0.5	—	587	1297	
1	Korovin	USA- AK	52.38	−174.15	198	160	—	1.2	0.5	—	163	148	
2	Langila	Papua New Guinea	−5.53	148.42	629	527	—	2.3*	0.7	—	994	886	*From the Group 2 global gas-rock association; Table S1d; Fig. 3a
2	Manam	Papua New Guinea	−4.08	145.04	1484	753	—	2.7*	0.7	—	2755	1570	*From the Group 2 global gas-rock association; Table S1d; Fig. 3a
1	Michael	South Sandwich Isl. (UK)	−57.80	−26.49	263	63	—	1.2	0.5	—	217	104	
1	Montagu	South Sandwich Isl. (UK)	−58.42	−26.33	142	179	—	1.2	0.5	—	117	155	
2	Paluweh	Indonesia/Nusa	−8.32	121.71	60	65	—	2.6*	1.3	—	108	130	*From the Sunda-Banda gas-rock association; Table S1c; Fig. 2g
2	Reventador	Ecuador	−0.08	−77.66	206	187	—	2.2*	0.8	—	304	298	*From the SA gas-rock association; Table S1b; Fig. 2d
3	Rinjani	Indonesia/Lombok	−8.42	116.47	74	131	—	4.3*	1.3	—	219	392	*From the Sunda-Banda gas-rock association; Table S1c; Fig. 2g
3	Sangeang Api	Indonesia/Nusa	−8.21	119.07	71	150	—	4.9*	1.3	—	239	508	*From the Sunda-Banda gas-rock association; Table S1c; Fig. 2g
1	Santiaguito	Guatemala	14.76	−91.55	247	119	—	1.6*	0.8	—	271	182	*From the CAVA gas-rock association; Table S1a; Fig. 2a
1	Sarychev	Russia	48.08	153.21	260	324	—	1.2	0.5	—	214	282	
2	Sinabung	Indonesia/Sumatra	3.17	98.39	327	595	—	2.4*	1.3	—	550	1043	*From the Sunda-Banda gas-rock association; Table S1c; Fig. 2g
2	Slamet	Indonesia/Java	−7.24	109.21	206	132	—	2.2*	1.3	—	311	272	*From the Sunda-Banda gas-rock association; Table S1c; Fig. 2g
2	Tavurvur	Papua New Guinea	−4.24	152.21	1729	2535	—	2.6*	0.7	—	3091	4607	*From the Group 2 global gas-rock association; Table S1d; Fig. 3a
2	Tinakula	Solomon back-arc	−10.38	165.80	256	276	—	2.1*	0.7	—	370	417	*From the Group 2 global gas-rock association; Table S1d; Fig. 3a
1	Tofua	Tonga Islands	−19.75	−175.07	284	89	—	1.2	0.5	—	235	122	
	Tungurahua	Ecuador	−1.47	−78.44	342	235	—	2.5*	0.8	—	588	445	*From the SA gas-rock association; Table S1b; Fig. 2d
2	Ulawun	Papua New Guinea	−5.05	151.33	630	581	—	2.4*	0.7	—	1040	1005	*From the Group 2 global gas-rock association; Table S1d; Fig. 3a
1	Veniaminof	USA- AK	56.17	−159.38	255	214	—	1.2	0.5	—	211	197	
					Measured SO_2_ flux	SD				Measured CO_2_ flux	Predicted CO_2_ flux	Total CO_2_ flux	
GRAND TOTAL (Mt/yr, 10^9^ kg/yr)					23	15				27.4 ± 3.6	11.4 ± 1.4	38.7 ± 2.9	
GRAND TOTAL (10^12^ mol/yr)					0.36	0.23				0.62 ± 0.08	0.26 ± 0.02	0.88 ± 0.06	

The quoted SO_2_ fluxes (column F) are 2005–2015 averages (and standard deviation, SD) taken from the compilation of ref.^30^. The “measured volcanoes” list includes those volcanoes for which SO_2_ flux and gas composition molar CO_2_/SO_2_ ratios have both been measured. Each volcano is assigned to a given Group (1–4) (column A) based on the original categorization of ref.^36^ (non-arc volcanoes are assigned to Group 4). Unless indicated (see references in column O), the measured CO_2_/SO_2_ ratios (column H) are from ref.^36^. For these strongly degassing volcanoes, we assume total S (S_T_; quoted in 36) equals to SO_2_; SO_2_ satellite detection for all these volcanoes implies high-emission temperatures and limited or no interaction with hydrothermal system (and thus trivial reduced S species, such as H_2_S). Marapi volcano in Sumatra is an exception because of its hydrothermal signature (high CO_2_/SO_2_, high H_2_S) and is not assigned to any specific group (N.D. = not determined). In cases where combined emissions from two volcanoes are listed in the original dataset^30^ (see volcanoes labelled with superscripts ^&^ and ^$^ in column B), due to insufficient spatial OMI resolution, a weighted average was calculated from the available volcanic gas information for the 2 where possible. Otherwise, equal^$^ gas contribution was assumed for the two volcanoes. The measured CO_2_ flux (column L) is calculated from the product of F by H (the quoted standard deviations in column N are based on propagation of the respective errors). The “unmeasured volcanoes” list includes volcanoes for which gas CO_2_/S_T_ data are unavailable. Thirteen of such “unmeasured” (for gas) volcanoes are sited in arc segments with no subducted carbonate-rich lithologies at the respective trenches, and are therefore assigned to Group 1 (e.g., they are assigned the mean CO_2_/S_T_ ratio of 1.2 ± 0.5 of Group 1 volcanoes; see Table S1). For the remaining volcanoes, we predict the time-averaged CO_2_/S_T_ (here considered as equivalent to CO_2_/SO_2_; column K) from the averaged (mean) trace-element composition of the corresponding volcanic rocks (Table S1) and the individual arc/global arc CO_2_/S_T_ vs. Ba/La associations (see Figs 2 and 3). Uncertainty in the predicted CO_2_/S_T_ ratios (column I) is the confidence interval calculated from the regression line and one standard deviation about the regression, and incorporates uncertainty/variability in “measured” gas CO_2_/S_T_ ratios (average uncertainty at 1σ, ~26%) and whole-rock Ba/La ratios (average uncertainty at 1 σ, ~16%) (see Table S1). Column O references the supplementary table (Table S1) detailing the specific CO_2_/S_T_ vs. Ba/La relation used. The SO_2_ flux GRAND TOTAL of 23 ± 15 Mt/yr is from ref.^32^. The GRAND TOTAL for measured, predicted and total CO_2_ flux is obtained by applying Monte Carlo method to the CO_2_ datasets of columns L, M and L + M, respectively. For each of the three datasets, 100 simulations are considered. In each simulation, the CO_2_ flux for each volcano is left to vary randomly within its mean ± SD value, and the resulting CO_2_ fluxes are summed up. The procedure is repeated 100 times, yielding 100 random-generated sums. The GRANDTOTAL values quoted in the tables are ranges (mean ± 1 SD) of 70% of the three populations of random-generated sums (e.g., the 15% outliers on each end of the populations are omitted). With this procedure, the global volcanic CO_2_ flux is assessed at 38.7 ± 2.9 Mt/yr, ~11.4 ± 1.4 Mt/yr of which is estimated for the 34 “unmeasured” volcanoes (those with no measured gas data available).

Here, we explore an alternative approach of indirectly inferring the CO_2_/S_T_ ratio signature of these “unmeasured” volcanoes, and ultimately their CO_2_ flux, based on the (far more commonly measured) trace element compositions of their erupted volcanic rocks. Volcanic arc gas CO_2_/S_T_ ratios and whole-rock trace element ratios (e.g., Ba/La or Sr/Nd ratios) are globally linked^36^, as both volatiles and fluid/melt-mobile elements (e.g., Ba and Sr) are sourced from fluids delivered from dehydration/melting of subducting slab sediments and altered ocean crust^37–42^. Based on their gas vs. whole-rock associations, arc volcanoes cluster into three Groups^36^. Group 1, which includes C-poor arc volcanoes (gas CO_2_/S_T_ ratios ≤2), are thought^36^ to be sourced by a mantle wedge source contaminated by C-poor slab fluids (derived from either terrigenous sediments or altered oceanic crust). Group 2 volcanoes are assumed to inherit their C-richer (2≤ CO_2_/S_T_ ratios ≤4) gas composition from incorporation into the mantle wedge of slab fluids derived from melting of carbonated sediments. Group 3 (CO_2_/S_T_ ratios >4), finally, includes C-rich arc gases, supporting the involvement of an additional crustal C contribution (de-carbonation/assimilation of upper crustal limestones^43,44^).

We here establish systematic gas vs. rock relationships at the scale of individual arc segments and/or groups of volcanoes. These relationships, once set, allow us to predict the CO_2/_S_T_ ratio for any volcano for which trace-element whole-rock information (but not gas composition) is available. Ultimately, using these predicted CO_2/_S_T_ ratios in tandem with available SO_2_ flux information^30^, we derive CO_2_ fluxes for all current top-degassing volcanoes and, by summation, a refined inventory of decadal (2005–2015) global CO_2_ emissions from subaerial volcanism.

## Results

### CO_2_ fluxes for the Earth’s best-studied volcanoes

Roughly ~62% of the 91 strongest volcanic SO_2_ sources globally^30^ have been characterised for both SO_2_ flux and (episodically) for volcanic gas compositions (Table 1). CO_2_ fluxes are thus obtained (see “Methods”) by pairing the OMI-based time-averaged 2005–2015 SO_2_ fluxes^30^ with the characteristic (mean) CO_2_/SO_2_ ratios in the corresponding high-temperature magmatic gases (data from ref.^36^ unless otherwise noted). The so-derived CO_2_ fluxes (Table 1) range from 28 to 15,800 tons/day, and are in reasonable agreement (typically within a factor ≤40%) with the CO_2_ fluxes estimated using ground-based SO_2_ flux measurements^11,15^. We estimate the cumulative CO_2_ flux from the 57 volcanic sources with “measured” gas compositions by applying a Monte Carlo method (see Table 1) to the dataset. The obtained cumulative “measured” flux is 27.4 ± 3.6 Mt/yr (or 0.62 ± 0.08·10^12^ mol/yr).

### Matching gas and whole-rock trace element compositions

Thirty-four top-ranking volcanic SO_2_ sources do not have gas compositional records (Table 1). We hereafter refer to such volcanoes without CO_2_/S_T_ information as “unmeasured” volcanoes.

**Figure 1:**
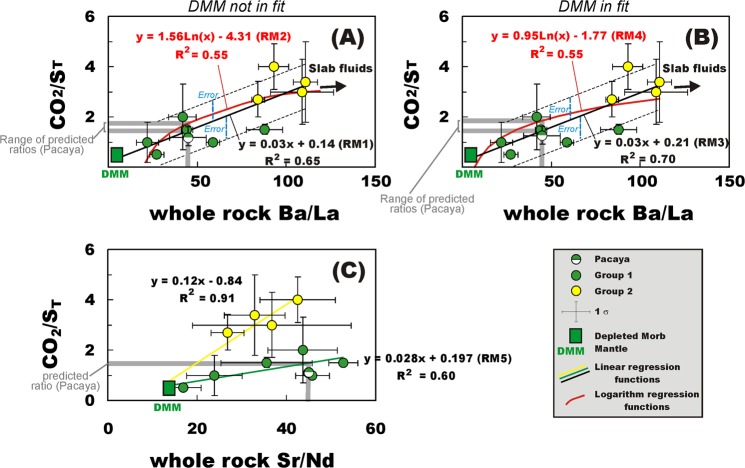
The proxy approach for estimating the CO_2_/S_T_ ratio for “unmeasured” volcanoes (i.e., those for which no gas data exist), based on the averaged trace-element composition of the corresponding volcanic rocks. The procedure is illustrated for Pacaya volcano in Guatemala. Firstly, the association between CO_2_/S_T_ ratios in volcanic gases (corresponding to CO_2_/SO_2_ gas ratios in the high-temperature systems studied here) and whole-rock Ba/La ratios is established at the scale of the Central American Volcanic Arc (CAVA) segments, using data for volcanoes for which both gas and trace element data are available (see Table S1a). Secondly, the gas vs. trace-element trend is fitted via either a linear or logarithm best-fit regression function. Tests made excluding (panel A) or including (panel B) the compositional point of the Depleted Mid-ocean ridge Mantle (DMM; refs^123,124^) in the data-fitting found that the second option systematically led to the best-data fits (see Table 2). Finally, the preferred regression model function (RM3 in the Pacaya example; see panel B and Table 2) is used to calculate a “predicted” gas CO_2_/SO_2_ from available Ba/La data for Pacaya whole-rocks (uncertainty is estimated from confidence interval at one standard deviation on the regression). Our inferred gas CO_2_/S_T_ ratio (1.4 ± 0.75; Table 2) is well within the magmatic gas range (CO_2_/SO_2_ ratio of 1.1 ± 1.0.) measured during recent plume observations^46^. A similar CO_2_/S_T_ ratio (see Table 2) is predicted using the CAVA gas vs. Sr/Nd ratio association (panel C). In this plot, the yellow and green dashed lines are the linear best-fit regression lines for Group 1 and 2 sub-populations, respectively.

**Figure 2:**
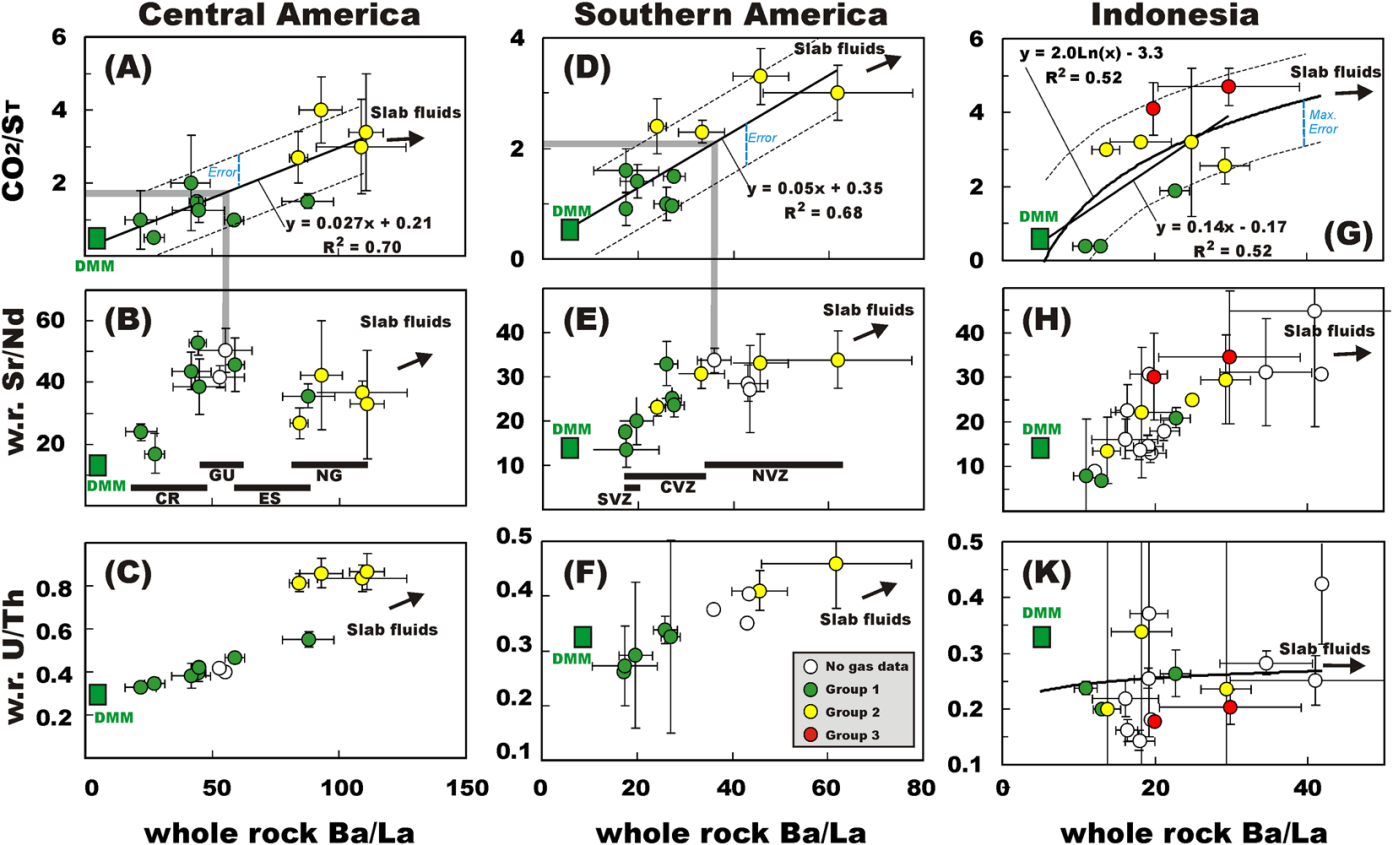
Scatter plots of mean Ba/La whole-rock ratios vs. volcanic gas CO_2_/S_T_ ratios (panels A, D and G), whole-rock Sr/Nd ratios (panels B, E and H) and whole-rock U/Th ratios (panels C, F and K) for three arc segments (left, Central America; middle, Southern America; right Sunda-Banda arc in Indonesia). Each symbol corresponds to an individual volcano for which gas and trace element information is simultaneously available (see Tables S1a–c for the list of volcanoes, compositions used, and data sources). The gas vs. trace element correlations are explained in terms of mixing between a C-Ba-Sr-U-poor Depleted Mid-ocean ridge Mantle (DMM) and C-Ba-Sr-U-rich slab fluids. C-poor arc volcanoes (Group 1, in green) plot close to the DMM, while Group 2 arc volcanoes (in yellow) are C-enriched to larger slab fluid influx. The even more C-rich signature of Group 3 arc volcanoes (in red) may reflect some addition of crustal carbon^36^. For each arc segment, panels A, D, and G show the best-fit regression functions used to predict the volcanic gas CO_2_/S_T_ ratios for “unmeasured” volcanoes (open symbols; see Tables 1 and S1b–d). The grey lines illustrate (for two “unmeasured” volcano examples) the procedure used to convert whole-rock Ba/La ratios into gas CO_2_/S_T_ ratios, using the equations of the best-fit regression lines.

**Figure 3:**
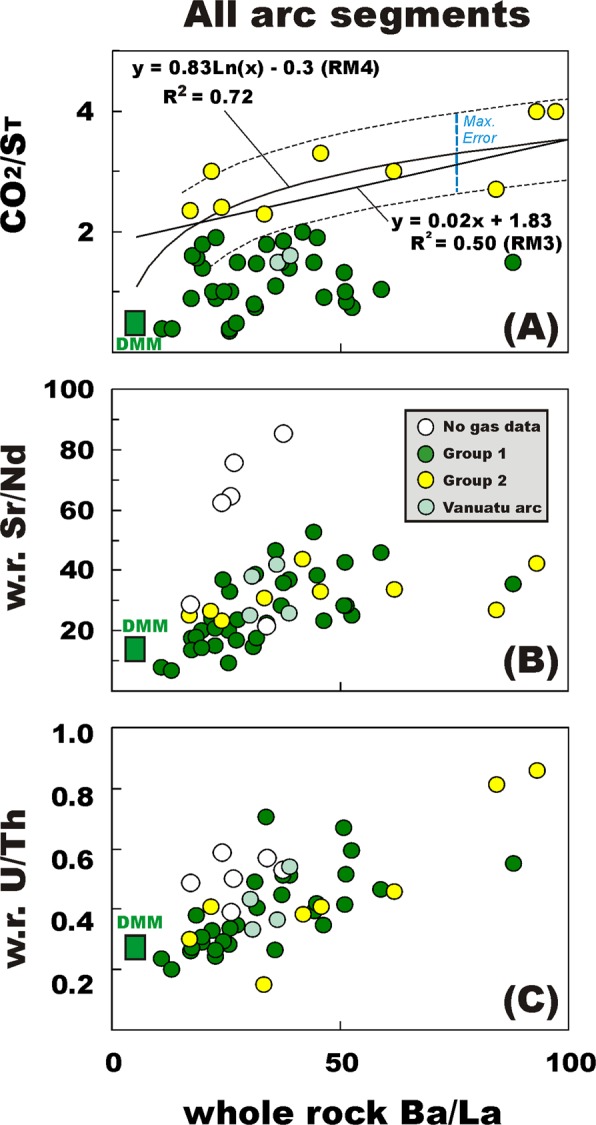
Scatter plots of mean Ba/La whole-rock ratios vs. (**A**) volcanic gas CO_2_/S_T_ ratios, (**B**) whole-rock Sr/Nd ratios and (**C**) whole-rock U/Th ratios (panels C, F and K) for Group 1 volcanoes (green, see Table S1d) and Group 2 volcanoes (yellow, see Table S1e) globally. Each symbol corresponds to an individual arc volcano for which gas and trace element information is simultaneously available (see Tables S1d–e for the list of volcanoes, compositions used, and data sources). Volcanoes with no gas compositional information are shown as open circles. The Vanuatu arc volcanoes are plotted in light green. The best-fit regression functions through the populations of Group 1 and Group 2 volcanoes are separately illustrated. Group 1 volcanoes exhibit little change in gas CO_2_/S_T_ ratios on increasing Ba/La. Their mean CO_2_/S_T_ ratio of 1.2 ± 0.5 (see Table S1d) is thus adopted for all the “unmeasured” (for gas) Group 1 volcanoes (Table 1). For the “unmeasured” Group 2 volcanoes, we average the predicted volcanic gas CO_2_/S_T_ ratios obtained from regression functions RM3 and RM4 (see Tables 1 and S1e).

We thus explore a methodology to predict the characteristic volcanic gas CO_2_/S_T_ ratio of each of these 34 “unmeasured” volcanoes using their averaged trace-element volcanic rock composition (Table S1). Gas CO_2/_S_T_ ratios in arc volcanoes exhibit systematic global relationships with slab fluid trace-element proxies (e.g., Ba/La or Sr/Nd ratios) in the corresponding whole-rocks, which are interpreted^36^ as resulting from a common CO_2_-Ba-Sr derivation from melting of subducted sediments in the slab^40^ (variably enriched in CO_2_; ref.^42^). These relationships, once set at the scale of individual arc segments (Figs 1 and 2) or volcano Groups (Fig. 3), can now be used to infer the representative volcanic gas CO_2_/S_T_ ratio signature of the 34 “unmeasured” volcanoes (Tables 1 and S1).

The procedure is illustrated in Fig. 1 and Table 2 for Pacaya volcano as an example (see “Methods”). The initial step involves establishing a CO_2_/S_T_ vs. Ba/La relationship using data for volcanoes for which both gas and trace element data are available (for the specific Pacaya example, we use gas/whole-rock information for Central American volcanoes, see Table S1a and Fig. 1). As in previous work^36^, the representative CO_2_/S_T_ ratios used in Fig. 1 (listed in Table 1 and S1a) are obtained by averaging available results for high-temperature gas samples, in the attempt to reduce the effect of secondary processes (e.g., magmatic S scrubbing during gas-water-rock reactions^45^) that become substantial at hydrothermal (temperature <400 °C) conditions. Secondly, regression analysis is used to fit the gas vs. trace-element association via either a (i) linear or (ii) logarithmic regression model (Fig. 1; see “Methods”). We focus on the two regression models based on the assumption that linear/logarithmic functions best describe depleted mantle (DMM)-slab fluid mixing in a CO_2_/S_T_ ratio vs. Ba/La (or Sr/Nd) compositional field^36^. Finally, the adopted regression function is used to derive a “predicted” gas CO_2_/S_T_ from available Ba/La whole rock data (Fig. 1). In the specific Pacaya example (Fig. 1 and Table 2), using a linear regression to fit the volcanic gas and DMM data-points (our RM3 regression model, see “Methods” and Table S1), the “predicted” gas CO_2_/SO_2_ ratio is 1.4 ± 0.75, well within the magmatic gas range (CO_2_/SO_2_ ratio of 1.1 ± 1.0) recently determined^46^ from plume measurements (Fig. 1).

**Table 2:** Comparison between measured^46^ and predicted (this work) volcanic gas CO_2_/S_T_ ratios in the Pacaya magmatic gases.

Measured CO_2_/SO_2_ (molar)	Predicted CO_2_/S_T_ (linear regression model RM1)	Predicted CO_2_/S_T_ (logarithm regression model RM2)	Predicted CO_2_/S_T_ (linear regression model RM3)	Predicted CO_2_/S_T_ (logarithm regression model RM4)	Predicted CO_2_/S_T_ (linear regression model RM5)
**1.1 ± 0.9**	1.4 ± 0.75	1.6 ± 0.8	**1.4 ± 0.75**	2.0 ± 0.8	1.3 ± 0.75

At the high-T magmatic gas conditions explored here, total S (S_T_) corresponds to SO_2_. The predicted CO_2_/S_T_ ratios are obtained from the mean Ba/La ratio (or Sr/Nd; see RM5) in Pacaya whole-rocks using the regression functions through the CO_2_/S_T_ vs. Ba/La (or Sr/Nd; see RM5) association for CAVA volcanoes (dataset listed in Table S1a). Five distinct regression functions are tested, being illustrated (with their corresponding equations and regression coefficients) in Fig. 1. RM1 and RM2 (Fig. 1a) use linear and logarithmic regression models, respectively, and do not include the composition of the Depleted mid-ocean ridge Mantle in the fit. Regression models RM3 and RM4 (Fig. 1b) are, respectively, linear and logarithmic, and the composition of the Depleted mid-ocean ridge Mantle is included in the fit. Regression models RM5 (Fig. 1c) uses linear regression functions through the CO_2_/S_T_ vs. Sr/Nd association for Group 1 CAVA volcanoes. The linear regression model RM3 yields the highest regression coefficient (R^2^ = 0.7; see Fig. 1b), and is thus adopted here.

### CO_2_/S_T_ ratios from individual-arc gas vs. trace-element relationships

Gas vs. rock (trace element composition) associations are initially analysed at the scale of individual arc segments, in the assumption that, at such regional scales, sources and transport pathways of volatiles and trace elements are relatively uniform. In truth, intra-arc variations in thickness, age, thermal properties and composition of the slab and overlying plate^47^, and in the composition of subducted sediments^42^, are large enough to impact the mechanisms of magma generation, and thus impart regional trends in volatile^48^ and trace element^49^ signatures of erupted magmas. Nonetheless, it is on these individual-arc trends that we rely below. Three arc segments have enough volcanoes measured for both gases and rocks to allow reliable gas vs. rock associations to be established (Fig. 2).

The Central American Volcanic Arc (CAVA) CO_2_/S_T_ vs. Ba/La relationship, obtained from results listed in Table S1a, is illustrated in Figs 1 and 2a. The systematic along-arc trace-element patterns in CAVA volcanic rocks^49^ (Fig. 2b,c) originate from changes in geometry, age, thermal regime and extent of serpentinization of the subducting Cocos plate slab^50^. As more recently found^36,51^, such trace-element variations correlate with those of CO_2_/S_T_ ratios in high-temperature magmatic CAVA gases. These correlations (e.g., Figs 1 and 2a) have been explained^36,51^ as resulting from the variable addition of C-Ba-Sr-rich fluids issuing from melting of limestone-bearing slab sediments, with the highest slab-fluid influx being observed in Nicaragua^52^, where magmatic gases consistently have C-rich (Group 2) affinity (Fig. 2a). In Costa Rica and El Salvador, magmatic gases are typically C-poorer^36,51^ (Group 1), in line with the lower slab affinity (and more depleted mantle-like signature) of trace-element ratios (Fig. 2). All the CAVA volcanic SO_2_ emitters of Table 1 have been measured for gas composition (at least for their CO_2_/S_T_ ratio), except for Guatemalan volcanoes Fuego and Santa Maria. We use the CAVA CO_2_/S_T_ vs. Ba/La association (of Fig. 2a) to fill this gap of knowledge. Using the RM3 regression model in tandem with mean whole-rock Ba/La ratios (Table S1a and Fig. 2a), we infer CO_2_/S_T_ ratios of respectively 1.7 ± 0.75 (Fuego) and 1.6 ± 0.75 (Santa Maria).

Our compilation (Table 1) shows that volcanic gas CO_2_/S_T_ data are available for the majority of the volcanic SO_2_ emitters in the Northern (NVZ), Central (CVZ) and Southern (SVZ) Volcanic Zones^53^ of the Andes (Southern America). Very limited gas information is available^54^ for Ecuadorian volcanoes, however, and here we use the CO_2_/S_T_ vs. Ba/La association (for South-America: Fig. 2d) to fill this knowledge gap. In the Andes, there is documented evidence in the literature for large along-arc variations in volcanic rock trace-element geochemistry^55–58^. Our partial whole-rock dataset, based on the subpopulation of Andean volcanoes listed in Table S1b, demonstrates an overall south-to-north increase in trace-element slab-fluid proxies (Ba/La, Sr/Nd and U/Th; Fig. 2e,f), from Copahue volcano in Argentina (SVZ) to Nevado de Ruiz in Colombia (NVZ). Importantly, the along-arc variations in the volcanic gas CO_2_/S_T_ ratio scale well with the trace-element variation patterns (Fig. 2d), again suggesting common source processes. The trace-element signature of the three most actively degassing volcanoes today in Ecuador, Tungurahua^59^, El Reventador^60^ and Cotopaxi^61^ (the latter not appearing in the 91 list of top degassing volcanoes^30^), places Ecuadorian magmatism in an intermediate position between Colombian volcanoes in the NVZ (the richest in Ba and Sr, but also CO_2_; Fig. 2d) and intermediate C-rich Peruvian volcanoes^62^ further to the south (in the CVZ). The mean Ba/La ratios, combined with the CO_2_/S_T_ vs. Ba/La linear regression model displayed in Fig. 2d, constrain the CO_2_/S_T_ ratio for Tungurahua and El Reventador at 2.5 ± 0.8 and 2.2 ± 0.8, respectively (see Table 1 and S1b). A consistent CO_2_/S_T_ ratio is inferred for Cotopaxi (2.5 ± 0.8).

**Figure 4:**
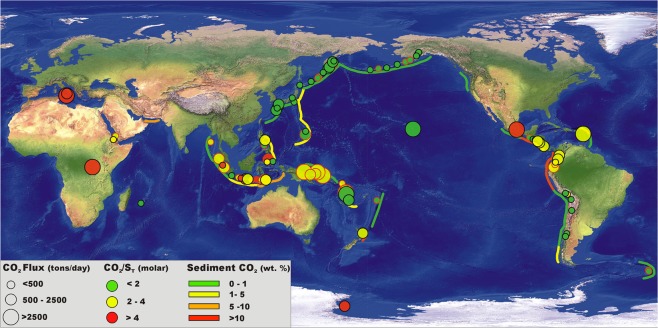
Global map illustrating the location of the 91 strongest volcanic CO_2_ emitters (data from Table 1). CO_2_ flux information for both “measured” (circles with black borders) and “unmeasured” (circles with red borders) volcanoes is shown. Dimension of the symbols is proportional to CO_2_ flux, with color fill reflecting the CO_2_/S_T_ ratio (see legend). Trenches are differently colored depending on CO_2_ bulk concentration in the trench sediments (data from ref.^42^). The map shows that the most strongly CO_2_ degassing volcanoes are clustered in tropic to sub-tropical regions such as the Vanuatu-Papua New Guinea arc segments, in Central America, Southern American (Northern Volcanic Zone), and in the Lesser Antilles, in addition to Italy (Etna), Congo (Nyrangongo + Nyamuragira) and Hawaii (Kilauea). Volcanic CO_2_ fluxes are typically lower in higher latitude volcanic regions such as in the Aleutians-Kamchatka-Kuriles and in the South-Sandwich Islands, where no carbonate-rich lithologies are subducted at the trenches. The map was generated using the open source QGIS software (available at https://www.qgis.org/it/site/) (Copyright © 2019 AIUPPA. Permission is granted to copy, distribute and/or modify this document under the terms of the GNU Free Documentation License, Version 1.3 or any later version published by the Free Software Foundation; with no Invariant Sections, no Front-Cover Texts, and no Back-Cover Texts. A copy of the license is included in the section entitled “GNU Free Documentation License”). The base map is a relief and bathymetry Raster called «Natural Earth II with Shaded Relief and Water» file #NE2_HR_LC_SR_W.tiff (Made with Natural Earth. Free vector and raster map data @ naturalearthdata.com). As for the shaded relief, we use the CleanTOPO2 layer, a modified SRTM30 Plus World Elevation Data also edited by Tom Patterson, US National Park Service. The original source data is from ref.^125^.

The case of Indonesia, which includes the Sunda-Banda and Sangihe-Halmahera arc segments, is particularly problematic (Fig. 2g–k). The large along- and within-arc variations in crustal^63^ and slab^64^ structures, combined with heterogeneities in the sedimentary slab input^42^ (Fig. 4), make it difficult to characterize regional trends in volatile sources. In the Java sector of the Sunda arc, the respective roles of crust and slab in controlling rock^65^ and gas^66^ geochemistry are widely debated, with some authors stressing the importance of upper plate assimilation^67,68^ and others emphasising a slab control^69–71^. The Group 3 signature^36^ of Merapi and Bromo (Fig. 2g) supports involvement of crustal carbon in Central Java^72^. South-to-north along-arc trends in gas ^3^He/^4^He (decreasing) and CO_2_/^3^He (increasing) ratios^66^ suggest a crustal volatile contribution is also likely in Sumatra, where the crust is especially thick and limestones widely exposed^63,67^. In contrast, crustal assimilation is supposedly minor (if any) in other sectors, including west and east-Java^65^, Nusa^69,73^ Banda^74^ and Halmahera^33^. In these segments of the Sunda-Banda and Sangihe-Halmahera arcs^75^, along-arc variations in He-C isotopes^66,76,77^, and the sparse high-temperature gas information, suggest variable C delivery from the slab, and thus coexistence of Group 1 and 2 volcanism (Fig. 2g). This is not unexpected, in view of the C heterogeneity in subducted sediments, from terrigenous and C-poor (Sumatra-Java) to pelagic and C-richer (Nusa, east Sunda)^42^ (Fig. 4). The diverse volatile sources that are possibly involved, in addition to the paucity of gas data, create scatter in CO_2_/S_T_ vs. Ba/La (Fig. 2g). Only 9 Indonesian volcanoes have been measured for both whole-rock trace element composition and (high-temperature) magmatic gas composition (Table S1c). These CO_2_/S_T_ vs. Ba/La data can be fitted by either a linear (RM3) or logarithm (RM4) regression model with identical regression coefficients (R^2^ = 0.52; Fig. 2g). We therefore infer the CO_2_/S_T_ ratio signature of the “unmeasured” Indonesian volcanoes (Table 1) by averaging the output of the two regression models (Table S1c). The low regression coefficients (Fig. 2g) imply the inferred CO_2_/S_T_ ratios should be treated with caution, as they require validation/refinement with an improved (more than 9 data-points) gas vs. trace element relationship. We caution, in particular, that the predicted CO_2_/S_T_ ratios (Table 1) may either over-estimate (for Group 1 volcanoes) or under-estimate (for Group 3 volcanoes) by a factor ~1.3 (the max error in Fig. 2g) the real volcanic gas CO_2_/S_T_ ratios of “unmeasured” Indonesian volcanoes.

### CO_2_/S_T_ ratios from Group-based gas vs. trace element relationships

Several of the “unmeasured” (for gas) volcanoes in Table 1 are sited in arc segments for which insufficient gas/rock information is currently available to establish individual-arc associations (as those analysed in Fig. 2). In order to derive information on their CO_2_/SO_2_ ratio gas signature, we use the global relationship between CO_2_/S_T_ and Ba/La in Groups 1–2 volcanoes (ref.^36^) (Fig. 3).

The majority of the remaining “unmeasured” (for gas) volcanoes in Table 1 are sited in arc segments for which available deep sea drill holes point to the lack of C-rich lithologies (limestones) in the subducted sediment succession^42^ (Fig. 4). Trench sediments poor in C have been identified in the segment of the Pacific Ring of Fire (Fig. 4) that stretches from Aleutians-Kuril-Kamchatka to the N/NW to Marianas/Japan/Philippines further south (10 “unmeasured” volcanoes in total – see Table 1). Where high-temperature gas information is available, a CO_2_-poor (Group 1) signature of volcanic gases^36^ has typically been observed in such carbonate-poor trenches (Fig. 4), matching well the small sedimentary slab C input^42^. Sediments are similarly C-poor (e.g., prevailingly terrigenous and biosiliceous^42^) in the Tonga and South Sandwich arcs (3 “unmeasured” volcanoes; Fig. 4). We therefore assign to Group 1 all the “unmeasured” (for gas) arc volcanoes fed by carbonate-poor trenches. Group 1 volcanoes exhibit little change in gas CO_2_/S_T_ ratios with increasing Ba/La (Fig. 3a). This implies either (i) limited C delivery from the slab in the absence of carbonated sediments (e.g., that fluids/melts delivered by terrigenous sediments, altered oceanic crust and/or serpentinite are not major C sources^36^), or (ii) that slab C and S are added to the mantle wedge in 1:1 to 4:1 proportions at most (Group 1 volcanoes typically have CO_2_/S_T_ ratios ~3–4 times higher than the DDM). The lack of dependence on Ba/La (Fig. 3a) means that we can prudently use the measured Group 1 CO_2_/S_T_ ratio average (1.2 ± 0.5; see Table S1d) for all the “unmeasured” (for gas) Group 1 volcanoes in Table 1.

Group 2 volcanoes are, by definition^36^, those having CaCO_3_-rich sediments in their trenches. These volcanoes typically have more C-rich volcanic gas composition (CO_2_/S_T_ ratio >2 but ≤4) and exhibit stronger, steeper correlation between gas CO_2_/S_T_ and trace element ratios (Fig. 3a). These Group 2 volcanoes are located in high biological productivity zones close to the tropics, where sediments are increasingly biogenic in nature and/or where seafloor is shallow enough (above the calcite compensation depth, CCD) to support carbonate deposition^42^ (Fig. 4). Of the few remaining “unmeasured” (for gas) volcanoes in Table 1, those in the Papua New Guinea-Solomon-Vanuatu arc segment are thus potential candidates for Group 2. The Papua New Guinea-Solomon arc sectors (Fig. 4) are a particular challenge because no gas samples are available, and no deep sea drill holes have been placed in the seafloor of the Solomon Sea, seaward of their trenches. Likewise, there are few relevant piston cores to provide any seafloor samples. Our inferences are thus based on seafloor depth, assumptions about the regional CCD, and drill sites in other, nearby southwest Pacific marginal seas. At DSDP Site 63, in the East Caroline Basin north of New Britain, carbonate lithologies were encountered throughout the entire section, from the Quaternary to the middle Oligocene basaltic basement^78^. This site, at 4472 m water depth, has thus been above the CCD over its entire history. Similarly, drilling at DSDP 287 (4653 m water depth), in the Coral Sea south of Papua New Guinea and east of the Solomon Islands, intercepted abundant carbonate lithologies through most of the sedimentary section to its lower Eocene basement^79^. Given that the water depths of the Solomon Sea are predominantly <4500 m seaward of the New Britain, Solomon and Northern Vanuatu trenches, we expect this seafloor to have been above the CCD for much of its history as well, and thus to be delivering carbonate-rich sediment to these subduction zones. Based on the above, we consider it very likely that “unmeasured” volcanoes in the Papua New Guinea- Northern Solomon-Vanuatu arcs belong to Group 2. We use therefore the CO_2_/S_T_ ratio vs. Ba/La global association for Group 2 volcanoes (see Fig. 3a) to predict (based on trace element information) CO_2_/S_T_ ratios ranging from 2.1 ± 0.7 to 2.7 ± 0.7 for these volcanoes (Tables 1 and S1e). We note that the two “measured” volcanoes in the central and southern Vanuatu arc (Bembow on Ambrym Island, and Yasur on Tanna Island) both exhibit Group 1 gas affinity (CO_2_/S_T_ of 1.5–1.6), implying that the predicted C-richer gas signature for northern Vanuatu volcanoes requires validation from measurements.

## Discussion

### Validity of whole rock trace element proxy for CO_2_/S_T_

Our predicted CO_2_/S_T_ ratios stand on the assumption that gas compositions are linked to trace element compositions of their source magmas at either regional (Fig. 2) or global (Fig. 3) scale. Implicit in establishing such relationships is that gas (CO_2_/S_T_) and trace-element (Ba/La) whole-rock tracers are inherited by the same processes at their source, and are similarly conserved during magma ascent, decompression and degassing/eruption^36^. For Ba/La, a link has been made between signatures of arc rocks and subducted sediments at corresponding tranches^80^, so that this and other trace element ratios are commonly used as slab-fluid proxies for charactering the mantle source of magmas^81,82^. Both elements exhibit incompatible behaviour during magma differentiation, so that the source-inherited ratios are essentially conserved during magma evolution, at least for the mafic to intermediate (andesitic) magma compositions considered here (as outlined in the Method).

The behaviour of volatile components CO_2_ and S is obviously complex during the generation and evolution of slab fluids and mantle-derived magmas^83^. Not only are slab sources and processes only partially understood for C and S^12,39^, but these volatile species will be selectively extracted from melt and partitioned into the vapour phase according to their melt solubilities (that dependent in a complex fashion on magma T-P-X-redox conditions), upon magma decompression and differentiation^84,85^. One may thus argue that degassing-related fractionations, for which abundant model^86–88^, experimental^89^ and observational^90^ evidence exists, act as to render the CO_2_/S_T_ ratios in both degassed melt (preserved in melt inclusions in phenocrysts) and exsolved vapour (discharged as volcanic gases) unrepresentative of the mantle source compositions, and thus unlinked^91^ to Ba/La or other trace element proxies.

Where sufficient data exists (e.g., Figs 2a,d and 3a), however, the CO_2_/S_T_ vs. Ba/La correlations appear systematic and statistically significant, and we consider unlikely that these associations are purely accidental. Our regional/global associations here, thus, implicate that the time-averaged CO_2_/S_T_ ratios of volcanic gases ultimately reflect the volatile ratios in the parental (un-degassed) melt, and in the mantle source. To reconcile this with the well-established degassing-driven CO_2_ vs. S_T_ fractionations, we observe that, at least at mafic systems, comparison between measured and modelled (from numerical simulation of magma degassing paths using volatile saturation codes^86–88^) gas CO_2_/S_T_ ratios typically imply equilibrium pressures (e.g., pressures of final gas-melt segregation) of 0.1–5 MPa during quiescent degassing activity^29,36,84,85,92^. Thus, at least during non-eruptive periods, during which the majority of the volcanic gas observations in the literature are taken, observations and models both indicate very shallow (a few hundred meters below the magma-air interface) gas segregation from the convecting feeding magmas^93,94^. If shallow closed-system degassing conditions^85,94^ prevail, then the magmatic gas phase released as volcanic gas during open-vent activity does represent an integral of volatiles exsolved from melt during most (P > 5 MPa) of the magma decompression path. This released magmatic gas is thus very similar in composition to the source and parental melt volatile signature, irrespective of its hydrous (for arc volcanoes) or more H_2_O-poor (for non-arc systems) nature^20,93^. The short-lived (days to weeks) pulses of CO_2_-rich gas, seen prior to eruption of mafic arc volcanoes^27–29,84,92^, imply somewhat deeper (typically, ~10–30 MPa) last gas-melt equilibration, but yet suggest closed-system is maintained up to rather shallow levels in a magmatic plumbing system, at least during quiescence. During basaltic explosive activity, deeper gas segregation is implied by gas observations^95,96^, but such eruptive degassing contributes only a minor fraction of the total degassing budget, which is dominated by passive emissions^93^.

**Figure 5:**
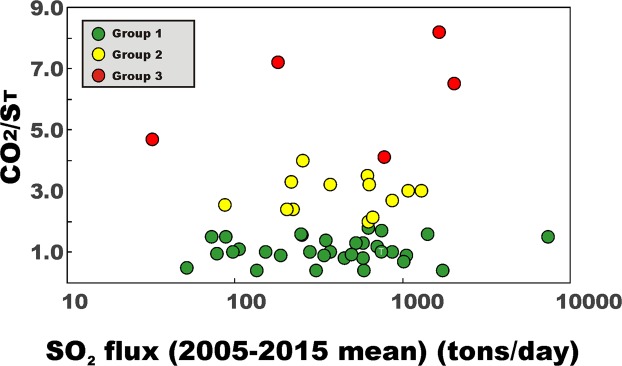
Scatter plot exploring the relationship between the SO_2_ flux (2005–2015 mean; data from ref.^33^) and the volcanic gas CO_2_/S_T_ ratio for the population of “measured” volcanoes in Table 1. For Turrialba + Poas (T), we plot the best-guess estimate for the magmatic gas CO_2_/S_T_ ratio for Turrialba volcano (data from 28 top-ranking volcanic point sources of SO_2_ (left) and CO_2_ (right) during 2005–2015. Data are from Table 1. SO_2_ fluxes are 2005–2015 means from ref.^30^. The CO_2_ fluxes are calculated from SO_2_ using measured or predicted CO_2_/SO_2_ ratios (see Table 1). Different volcano groups are identified by different colours. The global CO_2_ budget is dominated by CO_2_-rich Group 2–3 arc volcanoes. Two rift volcanoes (Nyiragongo and Nyamuragira) and one within-plate (WP) volcano (Kilauea) appears in the top-10 list of CO_2_ emitting volcanoes.

The lack of a systematic correlation between volcanic gas CO_2_/S_T_ ratios and SO_2_ fluxes (Fig. 5) further supports the idea that the former are not significantly affected by variable extents of magma degassing and gas-melt separation depths at various volcanoes. In mafic systems, the SO_2_ flux is a proxy for the rates of magma degassing in a volcano’s shallow (<3 km) plumbing system^93^. As such, at least in principle, shallow magma ascent and decompression should be tracked by increasing SO_2_ flux and decreasing CO_2_/S_T_ ratios in the surface gas output^26^, a relationship that is not observed in our global dataset (Fig. 5). The SO_2_ flux-independent, distinct CO_2_/S_T_ distributions of Group 1, 2 and 3 volcanoes (see Fig. 5) suggest, instead, that source signature, rather than degassing, ultimately controls the longer-term, time-averaged volcanic gas compositions. We caution that CO_2_/S_T_ ratio volcanic gas compositions may become less source-related in intermediate to silicic systems, where the gas output is often buffered by gas-melt equilibration in crustal, vapour-saturated magma reservoirs^97–100^. It is thus possible that part of the scatter in our gas vs. trace-element associations (Figs 2 and 3) is caused by the intermediate (andesitic) systems included in our dataset. Silicic systems have intentionally been excluded from our compilation.

The good match between our predicted and measured CO_2_/SO_2_ ratios at Pacaya volcano (Fig. 1) also support, although indirectly, the validity of our gas vs. trace element associations. In addition to Pacaya, recent airborne gas measurements^54^ at Tungurahua and Cotopaxi volcanoes in Ecuador have found CO_2_/SO_2_ ratios (in the 2 to 2.5 range) fully overlapping our predicted range (2.5 ± 0.8; Table 1). These successful tests provide confidence in the robustness of our predicted CO_2_/S_T_ ratios. We caution that, in order to validate our methodology further and reduce the scatter in gas vs. trace element scatter plots (e.g., Fig. 3g), gas observations should be prioritized in remote, unexplored volcanoes in Papua New Guinea, Sandwich Islands, Solomon Islands, Sumatra, east Sunda-Banda, and north-Vanuatu. In some of these arc segments (e.g., Sumatra, Sunda), crustal C may be involved^63,66,67^, in which case our predicted CO_2_/S_T_ ratios may underestimate the actual magmatic gas ratio (by a factor up to ~1.5–2). We also advise that, since only high-temperature (SO_2_-dominated) gas data are used to establish our gas vs. trace-element associations (Figs 2 and 3), our predicted CO_2_/S_T_ ratios are representative of the magmatic gas signature, irrespectively of whether or not hydrothermal processes are acting to alter the actual and total gas volcano emissions. For example, the hydrothermal (H_2_S-rich) gas emissions from Marapi volcano in Sumatra have measured CO_2_/S_T_ ratios of 20.5 ± 1.1 (Table 1), well distinct from what we would predict (CO_2_/S_T_ ratio of ~2.6) using the whole-rock Ba/La (19 ± 3; Table S1c) and the Indonesian gas vs. trace-element relationship (Fig. 2g). As such, discrepancy between measured and predicted CO_2_/S_T_ ratio at any other hydrothermal volcano may lead to apportioning the fraction of S lost to (or C produced by) the hydrothermal system. While we believe that hydrothermal processing should be the exception rather than the rule for the satellite-sensed volcanoes here, we ultimately anticipate our predicted CO_2_/S_T_ ratios (Table 1) will require revision and upgrading as new high quality gas data become available for newly measured volcanoes.

One important aspect to consider is that our regional/global associations (Figs 2 and 3) are based on averaging trace element information for rocks erupted during decades to millennia of volcanic activity. As such, the CO_2_/S_T_ ratios predicted from such associations should be viewed as long-term means over a volcano’s lifespan, rather than the instantaneous measurements as obtainable by direct gas observations. These “geologic” gas CO_2_/S_T_ ratios may thus serve, when combined with measured S content in mafic glass inclusions, to estimate the initial CO_2_ content in parental, un-degassed melts, and eventually in the sub-arc mantle. Both are similarly poorly contrained^101,102^ due to pre- and post-entrapment loss to vapour of poorly soluble CO_2_.

### A decadal global CO_2_ flux budget

**Figure 6:**
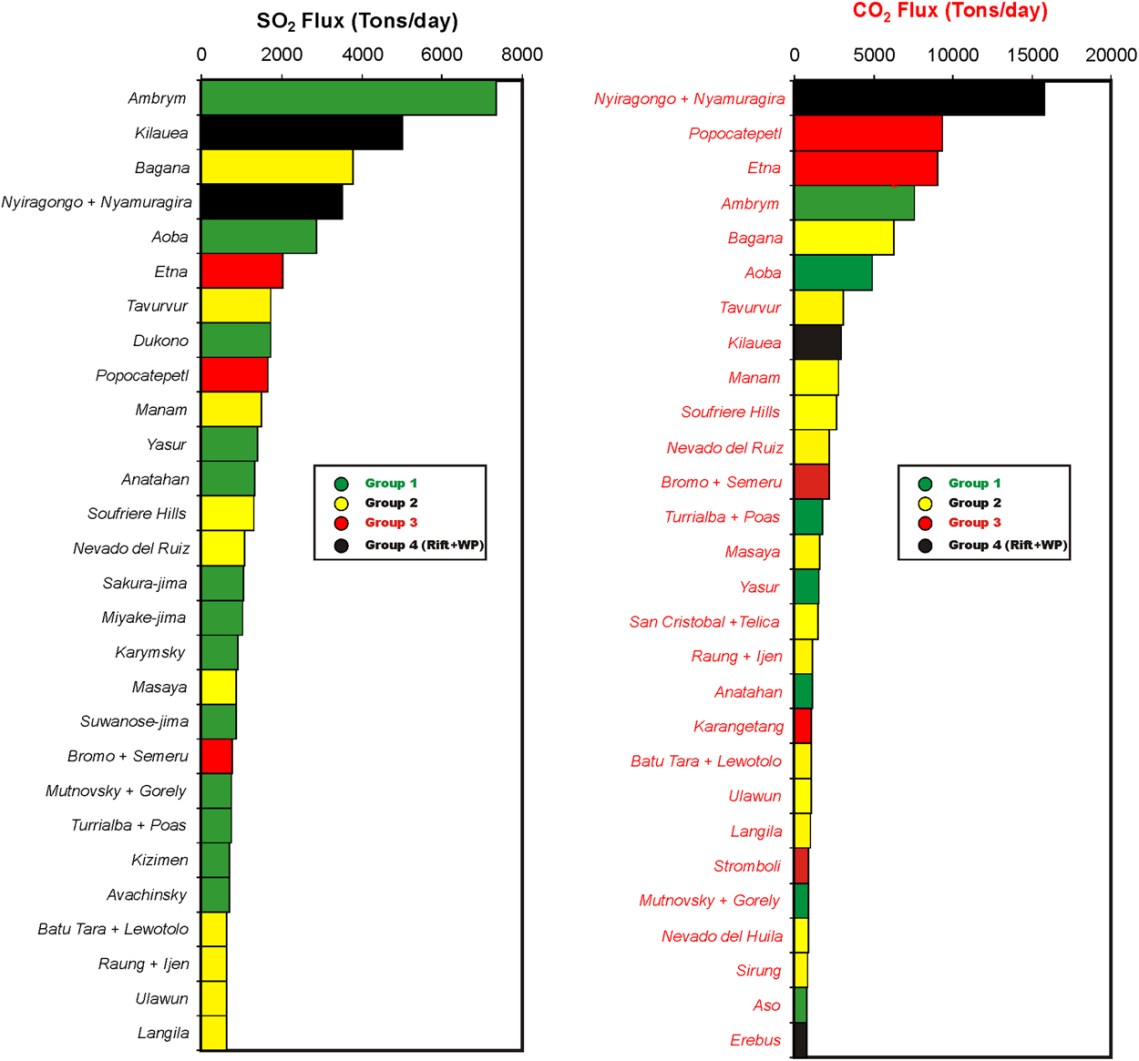
The 28 top-ranking volcanic point sources of SO_2_ (left) and CO_2_ (right) during 2005–2015. Data are from Table 1. SO_2_ fluxes are 2005–2015 means from ref.^30^. The CO_2_ fluxes are calculated from SO_2_ using measured or predicted CO_2_/SO_2_ ratios (see Table 1). Different volcano groups are identified by different colours. The global CO_2_ budget is dominated by CO_2_-rich Group 2–3 arc volcanoes. Two rift volcanoes (Nyiragongo and Nyamuragira) and one within-plate (WP) volcano (Kilauea) appears in the top-10 list of CO_2_ emitting volcanoes^110^.

Our predicted CO_2_/S_T_ ratios are converted into CO_2_ fluxes (Table 1) by assuming S_T_ = SO_2_ and scaling to the OMI-based mean SO_2_ fluxes for the 2005–2015 period^30^. We focus on the OMI satellite dataset owing to advantages brought by its global and coincident observations, but yet observe that quantitatively similar results would be obtained using ground-based SO_2_ flux observations instead^15^. The predicted CO_2_ fluxes range from 57 tons/day (Kanlaon volcano in the Philippines) to 6200 tons/day (Bagana volcano in PNG) (Figs 4 and 6). Uncertainty in the derived CO_2_ fluxes (see Table 1, column N) is based on propagation of the respective errors on SO_2_ flux (column G) and predicted CO_2_/S_T_ ratios (column I).

The total cumulative CO_2_ emissions from the 34 “unmeasured” volcanoes (those with no measured gas information available) would thus be ~11.4 ± 1.1 Mt/yr (~0.26 ± 0.02·10^12^ mol/yr), thus adding an additional ~34% to the cumulative “measured” mean CO_2_ emissions in 2005–2015 (27.4 ± 3.6 Mt/yr; Table 1). Finally, our extrapolated (measured + predicted) CO_2_ flux budget is 38.7 ± 2.9 Mt/yr (or 0.88 ± 0.06·10^12^ mol/yr). It is important to notice that our approach, in which the CO_2_/S_T_ ratio signature of each volcano is independently evaluated, leads to far better constrained CO_2_ budget (7% uncertainty at 1 SD) that would be possible using any “averaged” volcanic CO_2_/S_T_ ratio proxy (as has been often attempt in past studies). For example, scaling the mean global SO_2_ flux (23 ± 15 Mt/yr) to the mean volcanic CO_2_/S_T_ ratio (2.7 ± 3.6) (all data from Table 1) would lead to a global CO_2_ flux of 62 ± 92 Mt/yr (e.g., 148% uncertainty at 1 SD).

Based on our results, we infer that 6 strongly degassing volcanoes with time-averaged (2005–2015 means) CO_2_ fluxes of ≥ 5000 tons/day dominate the global CO_2_ budget (Figs 4 and 6). One of these (Bagana, PNG) is an “unmeasured” volcano and would not have been identified as a top CO_2_ emitter without the proxy approach developed here. It is interesting to observe that while the SO_2_ global budget is dominated by the Group 1 volcanoes (accounting for 13 out of the 28 strongest volcanic SO_2_ sources; Fig. 6), the CO_2_ global budget is predominantly determined by the CO_2_-enriched arc volcanoes in Group 2 (13 out of 28) and Group 3 (5 out of 28, with 2 - Popocatepetl and Etna - in the top-5 list) (Fig. 6). Two continental rift volcanoes (Nyiragongo and Nyamuragira) and two within-plate volcanoes (Kilauea and Erebus) also appear in our top-28 list of volcanic CO_2_ emitters (Fig. 6).

Our extrapolated global CO_2_ flux of 38.7 ± 2.9 Mt/yr is lower than previous global volcanic CO_2_ flux estimates in the literature, ranging from 66 to 540 Mt/yr (see ref.^11^ for a review). Several causes can explain this mismatch.

First, and most importantly, our global volcanic CO_2_ budget here only includes the contribution from the “strongly degassing volcanoes” that emit SO_2_ in quantities large enough to be detected from space (by OMI in this specific case^30^). We therefore admittedly do not take into consideration in our estimate the CO_2_ contribution from mildly degassing “magmatic” volcanoes (those still emitting SO_2_, but at levels too low to be resolved by satellites) and from “hydrothermal” volcanoes in which CO_2_ is emitted in combination with H_2_S (instead of SO_2_). Although typically exhibiting weaker surface gas manifestations, compared to the OMI-detected volcanoes characterised here, these magmatic-hydrothermal systems do often exhibit C-rich gas compositions^36^ (reflecting the extent/mechanism of gas-water-rock reaction with meteoric-hydrothermal fluids^45^), and do emit CO_2_ at the ~1000 tons/day level in the most extreme cases^17^, but most typically in the hundreds of tons/day range^15^. Considering that several hundreds of volcanoes worldwide are currently undergoing mild magmatic-hydrothermal degassing activity, this emission type could be responsible for the emission of several tens of Mt CO_2_/yr globally^11,15^. Also, we do not account for the CO_2_ output from volcanic lakes^103^, and diffuse/regional soil CO_2_ emissions around volcanic systems^104^, for which more data and alternative extrapolation approaches would be required. We therefore stress our results are not intended to represent total CO_2_ emissions from global subaerial volcanism, but rather the magmatic CO_2_ budget fraction contributed by the most actively degassing volcanoes on Earth.

Secondly, the mismatch in the estimated CO_2_ fluxes (this work and previous studies) derives (at least partially) from the distinct gas datasets used. We here specifically base our CO_2_ budget calculations on a consistent set of coincident (satellite-based) SO_2_ flux measurement, taken during a relatively short (decadal) period and with same retrieval/processing technique. In contrast, previous estimates have been hampered by the combination of sparse observations, taken over several decades, and with diverse observational/retrieval techniques. Even volcanoes which are persistently active alternate periods of elevated degassing with phases of reduced activity, and so non-coincident observations (taken over periods spanning several decades) may lead to biases. For example, by combining measurements taken between 1954 and 2011, a cumulative CO_2_ flux of 59.7 Mt/yr (from 33 measured volcanic gas plumes) was obtained^11^, or 2 times more than our mean 2005–2015 flux. We also explicitly use CO_2_/SO_2_ information for high-temperature magmatic gases only, in contrast with previous efforts^23^ in which individual arc CO_2_ emissions have been quantified also considering low-temperature hydrothermal gas samples in which the C-rich composition is not representative of the strongly degassing “magmatic” arc systems. We also cannot rule out that part of the discrepancy is due to our Ba/La approach, which may only represents the sub-Moho magmatic CO_2_ flux, and not a potentially large^44^ recycled crustal CO_2_ flux. Finally, our “measured” CO_2_ dataset is extrapolated to the total number of “unmeasured” strongly degassing volcanoes by predicting, for each of them, the specific CO_2_/SO_2_ ratio gas signature, rather than relying on the assumption that the global CO_2_ flux population obeys a specific statistical distribution (e.g., the power law distribution^105^).

Our results implicate that the arc volcano C flux (~8 ± 0.6 Mt C/yr) corresponds to a significant amount (~50%) of the subducted sedimentary carbonate (15 ± 2 Mt/yr; ref.^106^), but only a relatively small fraction ( < 21%) of the total C input at arc trenches (40–114 Mt C/yr; refs^1,12^). Thus, either the C input is balanced by “diffuse” C output forms, such as regional aquifers or soil degassing^107^ in the arc crust, or a substantial fraction of the subducted C is ultimately not erupted, but rather stored either in the lithospheric mantle^8^ or in the deep mantle^1,2^.

## Methods

The SO_2_ flux compilation^30^ we rely on in this study includes a list of the 91 top-ranking volcanic SO_2_ degassing sources in 2005–2015 (Table 1). This set of consistent (identical retrieval/processing technique) and simultaneous (global) measurements has improved upon the shortcomings of previous catalogues^108^, which combined SO_2_ fluxes obtained with diverse techniques and in disparate temporal intervals (often differing by several decades).

These SO_2_ flux data are converted into CO_2_ fluxes by using either measured or predicted molar CO_2_/S_T_ ratios. For these strongly degassing volcanoes, S_T_ is assumed to correspond to SO_2_ throughout, since SO_2_ detection by satellites implies limited or no interaction with hydrothermal system (and thus trivial reduced S species, such as H_2_S).

### Measured volcanoes

For 57 out of these 91 volcanic SO_2_ sources, we convert SO_2_ fluxes into CO_2_ fluxes, by pairing the former with the characteristic (mean) molar CO_2_/S_T_ (CO_2_/SO_2_) ratios in the corresponding volcanic gases (Table 1). For arc volcanoes, we use the time-averaged molar CO_2_/SO_2_ ratios compiled by (ref.^36^), integrated with novel gas information for eight new targets that have only recently been measured for the first time (see Table 1 for data provenance). Arc volcanoes are ranked in Groups (1 to 3) following the original categorization^36^. For non-arc volcanoes (here referred as Group 4), we average available volcanic gas information in the literature (see Table 1 for data sources). Note that, for both arc and non-arc, in cases where more than one volcano are listed in the original dataset^30^ (e.g., Nyiragongo + Nyamuragira) due to insufficient spatial OMI resolution, we averaged the available volcanic gas information for the individual volcanoes, weighting each volcano’s CO_2_/S_T_ ratio by its ground-based S flux (where available) to obtain a combined CO_2_/S_T_ ratio for the pair (see Table 1).

### Unmeasured volcanoes

Thirty-four out of the 91 top-ranking volcanic SO_2_ sources^30^ have never been characterised for volcanic gas composition (Table 1). These include four of the top-ten ranking volcanic SO_2_ emitters^30^ (Bagana, Rabaul and Manam in Papua New Guinea, and Aoba in the Vanuatu archipelagos; Fig. 4). To indirectly infer the molar CO_2_/S_T_ ratio gas signature of each of these 34 volcanoes, we use the averaged (mean) trace-element composition of the corresponding volcanic rocks. To this aim, as in earlier work^36^, we extract trace-element information (Ba, La, Sr, Nd, U and Th whole-rock concentrations) either from the Earthchem data-portal (http://www.earthchem.org/), or from other sources (for volcanoes that do not appear on Earthchem) (see Table S1). Mafic to intermediate (<55% SiO_2_) rocks are only considered, same as in other work^109^. From these, we calculate, for each volcano, the mean (±1 SD) of the Ba/La whole-rock ratios (Sr/Nd and U/Th ratios were also calculated; see Table S1). These ratios, in combination with the gas vs. whole-rock relationships illustrated in Figs 1–3, are finally used, to predict the characteristic volcanic gas CO_2_/S_T_ ratio signature for each of the 34 “unmeasured” volcanoes.

The procedure is exemplified in Fig. 1 for the Pacaya volcano example. We select Pacaya because the recently obtained gas compositions^46^ can serve as a test of the methodology. The initial step involves establishing the relationship between CO_2_/S_T_ gas ratios and whole-rock Ba/La ratios, using data for volcanoes for which both gas and trace element data are available (see Fig. 1; Table S1). The CO_2_/SO_2_ vs. Ba/La relationship can be established at the scale of individual arc segments (e.g., Figs 1 and 2), or for volcano Groups^36^ (Groups 1 or 2) (Fig. 3). For the Pacaya example, we rely on gas/whole-rock information for the well-characterised Central American Volcanic Arc (CAVA; Fig. 1). Secondly, we use regression analysis to fit the gas vs. trace-element association via either a (i) linear or (ii) logarithm regression model (Fig. 1). We find that linear regression yields the best data fit in the majority of the cases (see the Pacaya example, Fig. 1a,b), and this regression model is used throughout unless where indicated (see Table S1). We also find that data fitting is systemically optimised when the DMM composition is included in the fitting procedure (compare Fig. 1a,b), and this option is maintained throughout. Note, however, the method output (e.g., the outputted CO_2_/S_T_ ratio) is poorly sensitive to this choice (see Table 2). Finally, the adopted regression model function (RM3 in the Pacaya example; Fig. 1 and Table 2) is used to calculate a “predicted” gas CO_2_/S_T_ from available Ba/La information (Fig. 1). The confidence interval or delta, calculated from the regression line and one standard deviation about the regression, is taken as a proxy for the uncertainty in the predicted CO_2_/S_T_ ratios. Uncertainty on the predicted ratios, as derived, incorporates (although indirectly) uncertainty/variability in “measured” gas CO_2_/S_T_ ratios (average uncertainty at 1σ, ~26%) and whole-rock Ba/La ratios (average uncertainty at 1σ, ~16%) (see Table S1). In the specific Pacaya example (Fig. 1 and Table 2), our “predicted” gas CO_2_/SO_2_ ratio (1.4 ± 0.75) matches well the recently measured^45^ magmatic gas range (CO_2_/SO_2_ ratio of 1.1 ± 1.0). Our tests show that remarkably similar CO_2_/SO_2_ ratios (see Table 2) are obtained using other trace-element slab fluid tracers, such as the Sr/Nd ratio (Fig. 1c). We opt in the following for the Ba/La regression model because (i) La is more frequently available than Nd in the Earthchem dataset for the majority of the volcanoes, and (ii) use of the Sr/Nd ratio requires a priori knowledge of volcano affinity for a specific Group (Group 1 and 2 typically exhibit diverse distributions in a CO_2_/SO_2_ vs. Sr/Nd scatter plot; see Fig. 1c). This latter information is frequently not a priori available (see below). The same procedure is applied to all unmeasured volcanoes (Table S1a), and the “predicted” ratios (assumed to correspond to CO_2_/SO_2_) are combined with SO_2_ flux results to ultimately infer the CO_2_ fluxes (Table 1).

## Supplementary information

Supplementary Table S1
