# Supplementary material for: CO2 flux emissions from the Earth’s most actively degassing volcanoes, 2005–2015
Source: Sci Rep. 2019 Apr 1;9:5442. doi: 10.1038/s41598-019-41901-y (PMC6443792; doi:10.1038/s41598-019-41901-y)
Supplement: Supplementary file 1 — Supplementary Table S1 [file 41598_2019_41901_MOESM1_ESM.doc]

**Supplementary material for article**

**CO_2_ flux emissions from the Earth’s most actively**

**degassing volcanoes, 2005-2015**

Alessandro Aiuppa^1*^, Tobias P. Fischer^2^, Terry Plank^3^, and Philipson Bani^4^

*^1^Dipartimento DiSTeM, Università di Palermo, Italy*

*^2^Department of Earth and Planetary Sciences, New Mexico University, USA*

*^3^Lamont-Doherty Earth Observatory, Columbia University, USA*

*^4^Laboratoire Magmas et Volcans, Université Blaise Pascal - CNRS -IRD, OPGC, France*

**Supplementary Table S1** – Dataset used to establish the gas (CO_2_/S_T_ ratio) vs. trace-element associations illustrated in Figures 1-3, with respective data-sources. For the “unmeasured volcanoes” (those with no gas compositional information available), the predicted CO_2_/SO_2_ ratios are inferred from the listed Ba/La ratios and the gas CO_2_/S_T_ ratio vs. trace-element regression function (in view of the OMI-satellite SO_2_ detection, we assume S_T_ corresponds to SO_2_ in these strongly degassing volcanoes).

**S1a.** Measured gas compositions (CO_2_/S_T_ ratios) and trace-element ratios in erupted volcanics for CAVA volcanoes. These measured compositions are used to draw the scatter plots illustrated in Figure 1 and 2a. For unmeasured volcanoes, the predicted CO_2_/SO_2_ ratios are obtained from measured mean Ba/La ratios and the CAVA CO_2_/S_T_ ratio vs. Ba/La regression model RM3 (see equation in Figure 1b and 2a). The 95% confidence interval, calculated from the regression line at one standard deviation, is taken as a proxy for the uncertainty in the predicted CO_2_/S_T_ ratios. See table footnote for data source.

| **Central America** |  |  |  |  |  |  |  |  |  |
| --- | --- | --- | --- | --- | --- | --- | --- | --- | --- |
|  | Measured@  CO_2_/S_T_  (molar) | SD@ | Ba/La^#^ | SD^#^ | U/Th^#^ | SD^#^ | Sr/Nd^#^ | SD^#^ | Predicted^&^  CO_2_/SO_2_ (linear regression model RM3) |
| **DMM°** | 0.5 | 0.2 | 5 | - | 0.33 | - | 14 | - | - |
| **Turrialba** | 1.0 | 0.8 | 27.3 | 4.0 | 0.3 | 0.02 | 16.9 | 6.4 | - |
| **Poas** | 0.5 | 0.1 | 58.9 | 3.7 | 0.5 | 0.02 | 45.9 | 8.7 | - |
| **Santa Ana** | 1.0 | - | 44.3 | 3.3 | 0.4 | 0.04 | 52.8 | 3.9 | - |
| **Arenal** | 1.5 | - | 87.8 | 10.3 | 0.6 | 0.04 | 35.5 | 3.9 | - |
| **San Miguel** | 1.5 | 0.2 | 41.8 | 7.8 | 0.4 | 0.06 | 43.7 | 6.1 | - |
| **Colima** | 2.0 | 1.3 | 84.2 | 3.7 | 0.8 | 0.04 | 26.8 | 4.9 | - |
| **Masaya** | 2.7 | 0.7 | 109.0 | 17.8 | 0.8 | 0.06 | 36.7 | 3.7 | - |
| **Telica** | 3.0 | 1.3 | 111.0 | 6.8 | 0.9 | 0.09 | 32.9 | 17.6 | - |
| **Momotombo** | 3.4 | 1.6 | 93.0 | 8.4 | 0.9 | 0.07 | 42.5 | 17.7 | - |
| **San Cristobal** | 4.0 | 0.9 | 21.9 | 6.2 | 0.3 | 0.02 | 23.9 | 2.7 | - |
|  |  |  |  |  |  |  |  |  |  |
| **Pacaya** | 1.1 (0.5-1.5)^a^ | 0.9 | 44.9 | 10.2 | 0.42 | 0.02 | 38.5 | 9.0 | 1.4±0.75^&^ |
| **Fuego** | - | - | 55.2 | 10.6 | 0.40 | 0.02 | 50.4 | 7.0 | 1.7±0.75^&^ |
| **Santiaguito** | - | - | 52.8 | 9.7 | 0.42 | 0.08 | 41.8 | 3.5 | 1.6±0.75^&^ |
|  |  |  |  |  |  |  |  |  |  |

°Composition of the Depleted MOR Mantle from Saal et al., 2002 and Workman and Hart, 2005; @Time-averaged mean gas composition, taken from Aiuppa et al., 2017 except for ^a^Battaglia et al., 2018; ^#^Trace element information extracted from Aiuppa et al., 2017 and/or from the Earthchem data portal; ^&^Ratio and related error inferred from regression model function RM3, see Figure 2a.

**S1b.** Measured gas compositions (CO_2_/S_T_ ratios) and trace-element ratios in erupted volcanics for Southern American volcanoes. These measured compositions are used to draw the scatter plots illustrated in Figure 2d. For unmeasured volcanoes, the predicted CO_2_/SO_2_ ratios are obtained from measured mean Ba/La ratios and the Southern American CO_2_/S_T_ ratio vs. Ba/La regression model RM3 (see equation in Figure 2d). The 95% confidence interval, calculated from the regression line at one standard deviation, is taken as a proxy for the uncertainty in the predicted CO_2_/S_T_ ratios. See table footnote for data source.

| **Southern America** |  |  |  |  |  |  |  |  |  |
| --- | --- | --- | --- | --- | --- | --- | --- | --- | --- |
|  | Measured@  CO_2_/S_T_  (molar) | SD@ | Ba/La^#^ | SD^#^ | U/Th^#^ | SD^#^ | Sr/Nd^#^ | SD^#^ | Predicted^&^  CO_2_/SO_2_ (linear regression model RM3) |
| **DMM°** | 0.5 | 0.2 | 5 | - | 0.33 | - | 14 | - | - |
| **Copahue** | 0.9 | 0.3 | 17.3 | - | 0.3 | - | 17.6 | - | - |
| **Lastarria** | 1.6 | 0.4 | 17.5 | 6.9 | 0.3 | 0.1 | 13.6 | 4.1 | - |
| **Villarrica** | 1.0 | 0.3 | 25.9 | 2.5 | 0.3 | 0.0 | 33.0 | 5.0 | - |
| **Isluga** | 1.0^a^ | - | 27.1 | 2.0 | 0.3 | 0.2 | 25.1 | 2.5 | - |
| **Lascar** | 1.4 | 0.3 | 19.7 | 3.5 | 0.3 | 0.1 | 20.0 | 5.3 | - |
| **Nevado del Ruiz** | 3.0 | 0.5 | 61.8 | 15.7 | 0.5 | 0.1 | 33.8 | 6.5 | - |
| **Galeras** | 3.3 | 0.5 | 45.7 | 5.89 | 0.41 | 0.04 | 33.1 | 6.5 | - |
| **Ubinas** | 2.4 | 0.5 | 24.0 | 1.84 | - | - | 23.1 | 2.0 | - |
| **Sabancaya** | 1.5 | 0.1 | 27.5 | 2.26 | - | - | 23.6 | 2.8 | - |
| **El Misti** | 2.3 | 0.2 | 33.3 | 4.79 | - | 0.022 | 30.7 | 3.5 |  |
|  |  |  |  |  |  |  |  |  |  |
| **Tungurahua** | - | - | 43.1 | 4.2 | 0.4 | 0.3 | 28.5 | 4.1 | 2.5±0.8^&^ |
| **Reventador** | - | - | 36.0 | 3.5 | 0.4 | 0.0 | 33.7 | 2.8 | 2.2±0.8^&^ |
| **Cotopaxi** | - | - | 43.4 | 0.3 | 0.4 | 0.1 | 27.2 | 9.9 | 2.5±0.8^&^ |
|  |  |  |  |  |  |  |  |  |  |

°Composition of the Depleted MOR Mantle from Saal et al., 2002 and Workman and Hart, 2005;; @Time-averaged mean gas composition, taken from Aiuppa et al., 2017 except for ^a^Schipper et al., 2017; ^#^Trace element information extracted from Aiuppa et al., 2017 and/or from the Earthchem data portal; ^&^Ratio and related error inferred from regression model function RM3, see Figure 2d.

**S1c.** Measured gas compositions (CO_2_/S_T_ ratios) and trace-element ratios in erupted volcanics for Indonesian volcanoes (Sunda-Banda and Sangihe-Halmahera arcs. These measured compositions are used to draw the scatter plots illustrated in Figure 2g. For unmeasured volcanoes, the predicted CO_2_/SO_2_ ratios are obtained from measured mean Ba/La ratios and the Indonesian CO_2_/S_T_ ratio vs. Ba/La regression models RM3 and RM4 (see equations in Figure 2g). The 95% confidence interval, calculated from the regression line at one standard deviation, is taken as a proxy for the uncertainty in the predicted CO_2_/S_T_ ratios for RM3. For RM4, the error is taken as the maximum spread around the logarithm best-fit regression function. Outputs of RM3 and RM4 models are averaged (see last column), and these means are used in Table 1 to quantify the CO_2_ flux. See table footnote for data source.

| **Indonesia** |  |  |  |  |  |  |  |  |  |  |  |
| --- | --- | --- | --- | --- | --- | --- | --- | --- | --- | --- | --- |
|  | Measured@  CO_2_/S_T_  (molar) | SD@ | Ba/La^#^ | SD^#^ | U/Th^#^ | SD^#^ | Sr/Nd^#^ | SD^#^ | Predicted  CO_2_/SO_2_ (linear regression model RM3) | Predicted  CO_2_/SO_2_ (logarithm regression model RM4) | Predicted  CO_2_/SO_2_ (mean ) |
| **DMM°** | 0.5 | 0.2 | 5 | - | 0.33 | - | 14 | - | - | - | - |
| **Dukono** | 0.4 | 0.1 | 13 |  | 0.2 |  | 7 |  | - | - | - |
| **Lewotolo** | 1.9 |  | 23 | 2 | 0.3 | 0.04 | 21 | 2 | - | - | - |
| **Krakatau** | 0.4 |  | 11 | 2 | 0.2 | 0.01 | 8 | 13 | - | - | - |
| **Sirung** | 3.2^a^ | 2.0 | 25 | - | - | - | 25 | - | - | - | - |
| **Papandayan** | 3.0 | 0.1 | 14 | 2 | 0.2 | 0.01 | 14 | 7 | - | - | - |
| **Lokon-Empung** | 3.2^b^ |  | 18 | 4 | 0.3 | 0.21 | 22 | 15 | - | - | - |
| **Kawa-ijen** | 2.6^c^ | 0.5 | 29^$^ | 3^$^ | 0.2^$^ | 0.01^$^ | 29^$^ | 10^$^ | - | - | - |
| **Bromo** | 4.1 | 0.7 | 20 |  | 0.2 |  | 30 | 10 | - | - | - |
| **Merapi** | 4.7 | 0.5 | 30 | 9 | 0.2 | 0.03 | 34 | 15 | - | - | - |
| **Raung** | 3.3^d^ | 1.6 | - | - | - | - | - | - | - | - | - |
|  |  |  |  |  |  |  |  |  |  |  |  |
| **Barren Island** | - | - | 16 | 1.4 | 0.2 | 0.0 | 22 | 5.9 | 2.4±1.0^&^ | 2.1±1.0^&^ | 2.2±1.0^&^ |
| **Slamet** | - | - | 16 | 4 | 0.22 | 0.03 | 16 | 4 | 2.3±1.0^&^ | 2.1±1.0^&^ | 2.2±1.0^&^ |
| **Rinjani** | - | - | 35 | 6 | 0.28 | 0.02 | 31 | 12 | 3.9±1.0^&^ | 4.7±1.0^&^ | 4.3±1.0^&^ |
| **Ebulobo** | - | - | 19 | 2 | 0.37 | 0.22 | 31 | 1 | 2.7±1.0^&^ | 2.5±1.0^&^ | 2.6±1.0^&^ |
| **Sangeang Api** | - | - | 41 | 11 | 0.25 | 0.04 | 45 | 26 | 4.2±1.0^&^ | 5.6±1.0^&^ | 4.9±1.0^&^ |
| **Paluweh** | - | - | 19 | - | 0.18 | - | 13 | - | 2.7±1.0^&^ | 2.6±1.0^&^ | 2.6±1.0^&^ |
| **Karangetang** | - | - | 42 | - | 0.42 | 0.11 | 31 |  | 4.3±1.0^&^ | 5.7±1.0^&^ | 5.0±1.0^&^ |
| **Sinabung** | - | - | 18^£^ | 2^£^ | 0.14% | 0.002% | 15% | 1.2% | 2.6±1.0^&^ | 2.3±1.0^&^ | 2.4±1.0^&^ |
| **Kerinci** | - | - | 19^#^ | - | 0.22^#^ | - | 16^#^ | - | 2.7±1.0^&^ | 2.5±1.0^&^ | 2.6±1.0^&^ |
| **Marapi** | - | - | 19% | 3% | 0.24% | 0.03% | 15% | 6% | 1.8±1.0^&^ | 1.5±1.0^&^ | 1.6±1.0^&^ |
| **Batu tara** | - | - | 21^§^ | 3^§^ | - | - | 18^§^ | 2^§^ | 2.9±1.0^&^ | 2.8±1.0^&^ | 2.9±1.0^&^ |
|  |  |  |  |  |  |  |  |  |  |  |  |

°Composition of the Depleted MOR Mantle from Saal et al., 2002 and Workman and Hart, 2005;; @Time-averaged mean gas composition, taken from Aiuppa et al., 2017 except for: ^a^Bani et al., 2017; ^b^Bani et al., unpublished res.; ^c^Gunawan et al., 2016; ^d^Clor et al., 2005; ^#^Trace element information extracted from Aiuppa et al., 2017 and/or from the Earthchem data portal except for: ^$^Handley et al., 2007; ^§^Stolz et al., 1988; Van Bergen et al., 1990; Wheller et al. 1987; ^£^Nakada et al.,2017; %Bouvet de la Maisonneuve, pers. comm.; ^#^Tully et al.,2014; ^&^Error estimated from maximum data spread around the logarithmic regression model function RM4, see Figure 2g.

**S1d.** Measured gas compositions (CO_2_/S_T_ ratios) and trace-element ratios in erupted volcanics for Group 1 volcanoes globally. These measured compositions are used to draw the scatter plots illustrated in Figure 3 (green symbols). The mean CO_2_/S_T_ ratio for Group 1 arc volcanoes is evaluated at 1.2±0.5, and this ratio is assumed as representative of the gas CO_2_/SO_2_ ratios for all “unmeasured” Group 1 volcanoes worldwide (those for which no gas information is available, but are located in arc segments where CO_2_-poor sediments are being subducted at the corresponding trenches) (see Tab. 1).

| **Group 1@** |  |  |  |  |  |  |  |  |
| --- | --- | --- | --- | --- | --- | --- | --- | --- |
|  | Measured  CO_2_/S_T_  (molar) | SD | Ba/La | SD | U/Th | SD | Sr/Nd | SD |
| **Tokachi** | 0.4 | - | 25.6 | - | 0.3 | - | 20.0 | - |
| **Satsuma-Iwojima** | 0.4 | - | 25.6 | 1.7 |  | - | 9.3 | 0.1 |
| **Krakatau** | 0.4 | - | 10.9 | 1.5 | 0.24 | 0.01 | 7.8 | 12.7 |
| **Dukono** | 0.4 | 0.1 | 13.0 | - | 0.20 | - | 6.9 | - |
| **Poas** | 0.5 | 0.06 | 27.3 | 4.0 | 0.3 | 0.02 | 16.9 | 6.4 |
| **Miyake-jima** | 0.7 | - | 52.5 | 3.6 | 0.6 | 0.02 | 25.1 | 2.6 |
| **Pagan** | 0.8 | 0.0 | 31.4 | 7.7 | 0.5 | 0.1 | 38.8 | 19.7 |
| **Asama** | 0.8 | - | 31.1 | 9.9 |  | - | 14.8 | 5.0 |
| **Avacha** | 0.8 | - | 51.3 | 7.3 | 0.5 | 0.01 | 28.2 | 12.3 |
| **Copahue** | 0.9 | 0.3 | 17.3 | - | 0.3 | - | 17.6 | - |
| **Sakurajima** | 0.9 | - | 22.7 | 4.1 | 0.2 | 0.01 | 14.9 | 12.9 |
| **Kudriavy** | 0.9 | - | 46.38 | 15.5 | 0.35 | 0.03 | 23.4 | 8.1 |
| **Villarica** | 1.0 | 0.3 | 25.9 | 2.5 | 0.3 | 0.0 | 33.0 | 5.0 |
| **Turrialba** | 1.0 | 0.8 | 21.9 | 6.2 | 0.3 | 0.02 | 23.9 | 2.7 |
| **Redoubt** | 1.0 | 0.2 | 51.0 | 6.8 | 0.4 | 0.07 | 42.7 | 10.7 |
| **Suwanosejima** | 1.0 | - | 24.3 | 4.3 | 0.3 | 0.02 | 37.0 | 9.3 |
| **Santa Ana** | 1.0 | - | 58.9 | 3.7 | 0.5 | 0.0 | 45.9 | 8.7 |
| **Spurr** | 1.1 | - | 35.8 | 3.5 | 0.3 | 0.03 | 46.4 | 1.3 |
| **Kluichevskoi** | 1.3 | - | 50.8 | 11.0 | 0.7 | 0.1 | 28.2 | 7.4 |
| **Lascar** | 1.4 | 0.3 | 19.7 | 3.5 | 0.3 | 0.1 | 20.0 | 5.3 |
| **Iliamma** | 1.4 | 0.5 | 38.8 | 7.8 | 0.5 | 0.0 | 37.1 | 2.6 |
| **Gorely** | 1.5 | - | 31.66 | 2.2 | 0.41 | 0.01 | 17.4 | 11.6 |
| **Sabancaya** | 1.5 | 0.1 | 27.5 | 2.26 |  | - | 23.6 | 2.8 |
| **Arenal** | 1.5 | - | 44.3 | 3.3 | 0.4 | 0.04 | 52.8 | 3.9 |
| **San Miguel** | 1.5 | 0.2 | 87.8 | 10.3 | 0.55 | 0.04 | 35.5 | 3.9 |
| **Augustine** | 1.5 | 0.7 | 37.1 | 9.5 | 0.4 | - | 28.4 | 2.6 |
| **Ambrym (Bembow)** | 1.5 | 0.2 | 36.2 | 0.8 | 0.4 | 0.008 | 42.1 | 10.8 |
| **Ngauruhoe** | 1.6 | - | 18.5 | 1.3 | 0.4 | 0.07 | 18.0 | 3.4 |
| **Lastarria** | 1.6 | 0.4 | 17.5 | 6.9 | 0.3 | 0.1 | 13.6 | 4.1 |
| **Yasur** | 1.6 | 0.4 | 38.9 | 2.4 | 0.5 | 0.06 | 25.8 | 6.8 |
| **Tolbachik** | 1.8 | - | 33.8 | - | 0.7 | 0.1 | 22.7 | 8.2 |
| **Aso** | 1.8 | - | 19.7 | 2.6 | 0.3 | 0.03 | 14.5 | 2.5 |
| **Mutnovsky** | 1.9 | - | 37.51 | 4.8 | 0.51 | 0.06 | 35.7 | 11.4 |
| **Pacaya** | 1.9 | - | 44.9 | 10.2 | 0.4 | 0.02 | 38.5 | 9.0 |
| **Levotolo** | 1.9 | - | 22.7 | 2.0 | 0.26 | 0.04 | 20.9 | 2.2 |
| **Colima** | 2.0 | 1.3 | 41.8 | 7.8 | 0.4 | 0.1 | 43.7 | 6.1 |
| **Mean** | **1.2** | **0.5** | - | - | - | - | - | - |

@Time-averaged mean gas composition and trace element information extracted from Aiuppa et al., 2017

**S1e.** Measured gas compositions (CO_2_/S_T_ ratios) and trace-element ratios in erupted volcanics for Group 2 volcanoes globally. These measured compositions are used to draw the scatter plots illustrated in Figure 3 (yellow symbols). For unmeasured volcanoes, the predicted CO_2_/SO_2_ ratios are obtained from measured mean Ba/La ratios and the CO_2_/S_T_ ratio vs. Ba/La regression models RM3 and RM4 (see equations in Figure 3). The 95% confidence interval, calculated from the regression line at one standard deviation, is taken as a proxy for the uncertainty in the predicted CO_2_/S_T_ ratios for RM3. For RM4, the error is taken as the maximum spread around the logarithm best-fit regression function. Outputs of RM3 and RM4 models are averaged (see last column), and these means are used in Table 1 to quantify the CO_2_ flux. See table footnote for data source.

| **Group 2** |  |  |  |  |  |  |  |  |  |  |  |
| --- | --- | --- | --- | --- | --- | --- | --- | --- | --- | --- | --- |
|  | Measured@  CO_2_/S_T_  (molar) | SD@ | Ba/La^#^ | SD^#^ | U/Th^#^ | SD^#^ | Sr/Nd^#^ | SD^#^ | Predicted  CO_2_/SO_2_ (linear regression model RM3) | Predicted  CO_2_/SO_2_ (logarithm regression model RM4) | Predicted  CO_2_/SO_2_ (mean ) |
| **DMM°** | 0.5 | 0.2 | 5 | - | 0.33 | - | 14 | - | - | - | - |
| **El Misti** | 2.3 | 0.2 | 33 | 4.79 | 0.2 | 0.022 | 30.7 | 3.5 | - | - | - |
| **Soufriere, Guadeloupe** | 2.3 | - | 17 | 2.07 | 0.3 | 0.03 | 25.1 | 8.9 | - | - | - |
| **Ubinas** | 2.4 | 0.5 | 24 | 1.84 |  | - | 23.1 | 2.0 | - | - | - |
| **Masaya** | 2.7 | 0.7 | 84 | 3.7 | 0.8 | 0.04 | 26.8 | 4.9 | - | - | - |
| **Nevado de Riuz** | 3.0 | 0.5 | 62 | 15.7 | 0.5 | 0.1 | 33.8 | 6.5 | - | - | - |
| **Telica** | 3.0 | 1.3 | 109 | 17.8 | 0.8 | 0.06 | 36.7 | 3.7 | - | - | - |
| **Soufriere Hills, Montserrat** | 3.0 | 1.1 | 22 | 6.0 | 0.4 | 0.09 | 26.5 | 12.3 | - | - | - |
| **Padandayan** | 3.0 | 0.1 | 14 | 2.5 | 0.20 | 0.01 | 13.6 | 7.5 | - | - | - |
| **Galeras** | 3.3 | 0.5 | 45.7 | 5.89 | 0.41 | 0.04 | 33.1 | 6.5 | - | - | - |
| **Momotombo** | 3.4 | 1.6 | 111 | 6.8 | 0.9 | 0.09 | 32.9 | 17.6 | - | - | - |
| **San Cristobal** | 4.0 | 0.9 | 93 | 8.4 | 0.9 | 0.07 | 42.5 | 17.7 | - | - | - |
| **White Island** | 4.0 | - | 97 | 25.9 | 0.3 | 0.02 | 33.0 | 3.6 | - | - | - |
| **Mean** | **3.0** | **0.6** |  |  |  |  |  |  |  |  |  |
|  |  |  |  |  |  |  |  |  |  |  |  |
| **Bagana** | - | - | 26.0 | 4.4 | 0.4 | 0.06 | 64.4 | 10.8 | 2.3±0.7^&^ | 2.4±0.7^&^ | 2.4±0.7^&^ |
| **Manam** | - | - | 37.5 | 9.8 | 0.5 | 0.04 | 85.3 | 15.6 | 2.6±0.7^&^ | 2.7±0.7^&^ | 2.7±0.7^&^ |
| **Langila** | - | - | 24.1 | 2.8 | 0.6 | 0.03 | 62.4 | 6.0 | 2.3±0.7^&^ | 2.3±0.7^&^ | 2.3±0.7^&^ |
| **Ulawun** | - | - | 26.7 | 10.0 | 0.5 | 0.40 | 75.5 | 20.0 | 2.4±0.7^&^ | 2.4±0.7^&^ | 2.4±0.7^&^ |
| **Rabaul** | - | - | 34.0 | 12.8 | 0.6 | 0.04 | 21.5 | 12.8 | 2.5±0.7^&^ | 2.6±0.7^&^ | 2.6±0.7^&^ |
| **Tinakula** | - | - | 17.1^£^ | 2.3^£^ | 0.5^£^ | 0.12 | 28.6^£^ | 5.3^£^ | 2.2±0.7^&^ | 2.1±0.7^&^ | 2.1±0.7^&^ |
| **Aoba** | - | - | 30.8 | 6.0 | 0.3 | 0.01 | 38.1 | 9.0 | 2.4±0.7^&^ | 2.5±0.7^&^ | 2.5±0.7^&^ |
| **Gaua** | - | - | 30.3 | 5.3 | 0.4 | 0.06 | 25.0 | 9.6 | 2.4±0.7^&^ | 2.5±0.7^&^ | 2.5±0.7^&^ |
|  |  |  |  |  |  |  |  |  |  |  |  |

°Composition of the Depleted MOR Mantle from Saal et al., 2002 and Workman and Hart, 2005;; @Time-averaged mean gas composition, taken from Aiuppa et al., 2017; ^#^Trace element information extracted from Aiuppa et al., 2017 and/or from the Earthchem data portal, except ^£^Schuth et al., (2009); ^&^Error estimated from maximum data spread around the logarithmic regression model function RM4, see Figure 4a.

**References cited**

Aiuppa, A., Fischer, T.B., Plank, T., Robidoux P., Di Napoli, R. Along-arc, inter-arc and arc-to-arc variations in volcanic gas CO_2_/ST ratios reveal dual source of carbon in arc volcanism. *Earth Sci. Rev.* **168**, 24–47 (2017).

Bani, P., et al. First study of the heat and gas budget for Sirung volcano, Indonesia. *Bull. Volcanol.* **79:60**, DOI 10.1007/s00445-017-1142-8 (2017)

Battaglia, A., Bitetto, M., Aiuppa, A., Rizzo, A.L., Chigna, G., Watson, I. M., et al. The magmatic gas signature of Pacaya Volcano, with implications for the volcanic CO2 ﬂux from Guatemala. *Geochem. Geophys., Geosys*. **19**, 667–692, <https://doi.org/10.1002/2017GC007238> (2018).

Clor, L.E., Fischer, T.P., Hilton, D.R., Sharp, Z.D., Hartono, U. Volatile and N isotope chemistry of the Molucca Sea collision zone: Tracing source components along the Sangihe Arc, Indonesia. *Geochem. Geophys. Geosys*. **6** (3), art. no. Q03J14, DOI: 10.1029/2004GC000825 (2005)

Gunawan, H., et al., New insights into Kawah Ijen’s volcanic system from the wet volcano workshop experiment. In: Ohba, T., Capaccioni, B. & Caudron, C. (eds), *Geochemistry and Geophysics of Active Volcanic Lakes*. Geological Society, London, Special Publications, 437, <http://doi.org/10.1144/SP437.7> (2016)

Handley, H.K., Macpherson, G., Davidson, J.P., Berlo, K., Lowry, D. Constraining Fluid and Sediment Contributions to Subduction-Related Magmatism in Indonesia: Ijen Volcanic Complex. *J. Petrol.* **48**:6, 1155-1183, doi:10.1093/petrology/egm013 (2007)

Nakada, S., et al. Growth process of the lava dome/flow complex at Sinabung Volcano during 2013-2016. *J. Volcanol. Geotherm. Res*. https://doi.org/10.1016/j.jvolgeores.2017.06.012 (2017).

Saal, A.E., Hauri, E., Langmuir, C.H. and Perfit, M.R. Vapour under-saturation in primitive mid-ocean-ridge basalt and the volatile content of Earth’s upper mantle. *Nature* **419**, 451-455 (2002).

Schipper, C.I., et al. Isotopically (δ^13^C and δ^18^O) heavy volcanic plumes from Central Andean volcanoes: a field study. *Bull Volcanol* **79**, 65 DOI 10.1007/s00445-017-1146-4 (2017)

Schuth, S., Munker, C., Konig, S., Qopoto, C., Basi, S., Garbe-Schonberg, D., Ballhaus, C., Petrogenesis of Lavas along the Solomon Island Arc, SW Pacific: Coupling of Compositional Variations and Subduction Zone Geometry *J. Petrol.* **50:5**, 781-811 (2009)

Stolz, A.J., Varne, R., Wheller, G.E., Foden, J.D., Abbott, M.J. The geochemistry and petrogenesis of K-rich alkaline volcanics from the Batu Tara volcano, eastern Sunda arc. *Contrib. Mineral. Petrol*. **98**, 374-389 (1988)

Tully, M., Saunders, K., Troll, V. R., et al. Petrographic and Geochemical Investigation of Andesitic Arc Volcanism: Mount Kerinci, Sunda Arc, Indonesia. Proc. American Geophysical Union, Fall Meeting 2014, abstract id. V33B-4866 (2014)

van Bergen, M.J., Vroon, P.Z., Varekamp J.C., and Poorter R.P.E. The origin of the potassic rock suite from Batu Tara volcano (East Sunda Arc, Indonesia). *Lithos* **28**, 261-282 (1992).

Wheller, G.E., Varne, R., Foden, J.D., Abbott, M.J. Geochemistry of Quaternary volcanism in the Sunda-Banda Arc, Indonesia, and three-component genesis of island arc basaltic magmas. *J. Volcanol. Geotherm. Res*. **32**, 137-160 (1987).

Workman, R.K. Hart, S.R. Major and trace element composition of the depleted MORB mantle (DMM). *Earth Planet. Sci. Lett*. **231**, 53-7 (2005).
